# Neuroimmune Consequences of eIF4E Phosphorylation on Chemotherapy-Induced Peripheral Neuropathy

**DOI:** 10.3389/fimmu.2021.642420

**Published:** 2021-04-12

**Authors:** Nilesh M. Agalave, Prapti H. Mody, Thomas A. Szabo-Pardi, Han S. Jeong, Michael D. Burton

**Affiliations:** Neuroimmunology and Behavior Laboratory, Department of Neuroscience, School of Behavioral and Brain Sciences, Center for Advanced Pain Studies, University of Texas at Dallas, Richardson, TX, United States

**Keywords:** Dorsal Root Ganglia, astrocyte, microglia, T-cell, mitochondrial respiration, eIF4E, sex differences, neuroimmune

## Abstract

Chemotherapy-induced peripheral neuropathy (CIPN) is a major dose-limiting side effect that occurs in up to 63% of patients and has no known effective treatment. A majority of studies do not effectively assess sex differences in the onset and persistence of CIPN. Here we investigated the onset of CIPN, a point of therapeutic intervention where we may limit, or even prevent the development of CIPN. We hypothesized that cap-dependent translation mechanisms are important in early CIPN development and the bi-directional crosstalk between immune cells and nociceptors plays a complementary role to CIPN establishment and sex differences observed. In this study, we used wild type and eIF4E-mutant mice of both sexes to investigate the role of cap-dependent translation and the contribution of immune cells and nociceptors in the periphery and glia in the spinal cord during paclitaxel-induced peripheral neuropathy. We found that systemically administered paclitaxel induces pain-like behaviors in both sexes, increases helper T-lymphocytes, downregulates cytotoxic T-lymphocytes, and increases mitochondrial dysfunction in dorsal root ganglia neurons; all of which is eIF4E-dependent in both sexes. We identified a robust paclitaxel-induced, eIF4E-dependent increase in spinal astrocyte immunoreactivity in males, but not females. Taken together, our data reveals that cap-dependent translation may be a key pathway that presents relevant therapeutic targets during the early phase of CIPN. By targeting the eIF4E complex, we may reduce or reverse the negative effects associated with chemotherapeutic treatments.

## Introduction

Chemotherapy-induced peripheral neuropathy (CIPN) is one of the most prevalent and dose-limiting complications in chemotherapy patients. It is characterized by pain and numbness or a “pins and needles” feeling in the extremities (1–4). Approximately 70% of patients report the development of peripheral neuropathy during administration of chemotherapy, which leads to early treatment cessation and substandard regimens (5, 6). Additionally, over 30% of patients experience persistent neuropathy for months or years after treatment cessation, making CIPN a long-term morbidity in patients that survive cancer and other diseases (7).

There is limited data on sex differences in neuroimmune effects during CIPN. Both clinical and preclinical investigations have reported conflicting observations ranging from marked sexual dimorphisms to no sex differences during CIPN (8–11). There are few studies that investigate sex differences and the mechanisms involved in the development of CIPN. These findings necessitate a comprehensive and fully powered assessment of sex differences in the development of CIPN. Considering the recent interest in sex differences in chronic pain development and immune cell activation, it is purported that neuroimmune mechanisms vary between sexes. A benchmark paper in the pain field suggests sexual dimorphisms in the role of T-cells in pain initiation in females (12). Moreover, recent studies have shown that cytotoxic T-cells (CD8^+^) contribute to the resolution of CIPN (13, 14). A more recent study showed that “primed” CD8^+^ T-cells, previously exposed to a chemotherapy agent, could prevent CIPN development when transferred to a new host (15). Based on these previous studies, we decided to assess the role of discrete T-cell subtypes: CD4^+^(Th1, Th2), T regulatory (T_regs_), Effector (T_eff_), and activated cytotoxic T-lymphocytes (CD8^+^) in the draining lymph nodes of male and female mice after induction of CIPN.

Immune cells recruited to the dorsal root ganglion (DRG) in turn can influence the activity of nociceptors through the release of inflammatory mediators (16, 17). This signaling could happen in tandem, in real-time, and this bi-directional crosstalk could be directly responsible for changes in nociceptors and immune cells, leading to changes in pain behaviors. These reciprocal interactions may contribute to maladaptive nociceptor plasticity if the homeostatic functioning of the cell-mediated immune response is dysregulated and has been shown to contribute to the development of CIPN (18–21)

Eukaryotic translation initiation factor 4E (eIF4E) is a cytosolic regulator that directs ribosomes to the cap structure of a subset of mRNAs to induce translation of proteins involved in inflammation and pain (22, 23). Cellular signaling cascades responsive to external stimuli such as the mitogen-associated protein kinase (MAPK) pathway and the mammalian target of rapamycin (mTOR) pathway directly feed into central cap-dependent translation in all cell types (24, 25). Regulation of eIF4E activation affects downstream signaling for production of a subset of pro-inflammatory cytokines involved in T-cell subsets activation, cellular metabolic pathways of nociceptors, and other components that regulate pain, inflammation, and behavior (26–33). While we have begun to assess the role of eIF4E phosphorylation in sensory neuron populations (34), a complete breakdown of sex and sensory neuron metabolic phenotyping has yet to occur along with phenotyping of immune cell subtypes during the development of CIPN. It is known that sensory neuron metabolism is exceptionally responsive to nerve injury and inflammation (35–37). CIPN is accompanied with changes in mitochondrial bioenergetics and an energy deficit in DRG neurons (38, 39). These changes can also alter ion signaling and other downstream metabolic processes to contribute to long-term development of nociceptor sensitivity and pain (40). The metabolic effect(s) paclitaxel has in late CIPN, has been assessed in sensory neurons from male rats only (38).

Therefore, we sought to elucidate the role of cap-dependent protein translation in CIPN-induced alterations that may be mediated by neuroimmune communication between activated immune cells and sensory neurons. We used eIF4E mice that harbor a point mutation at the serine 209 site on the eIF4E complex that codes alanine instead, designated as eIF4E^S209A^ (41). This point mutation prevents phosphorylation of eIF4E, thus inhibiting its activation and consequently inhibiting cap-dependent protein translation (42, 43). Paclitaxel is one of many chemotherapeutics used in rodent models to mimic clinical CIPN (44). Given that CIPN has an elusive etiology and the diverse array of cell types that could participate in CIPN development, we investigated whether there was a sex-dependent role of eIF4E phosphorylation in DRG sensory neuron metabolism, local immune cell populations, peripheral T-cell subpopulations, and spinal microglia and astrocytes. We hypothesized that eIF4E phosphorylation regulates activation of specific macrophage and T-cell subpopulations, alters nociceptor metabolism, and glial activation during the early phase of paclitaxel-induced peripheral neuropathy.

## Materials and Methods

### Animals

**Figure 1:**
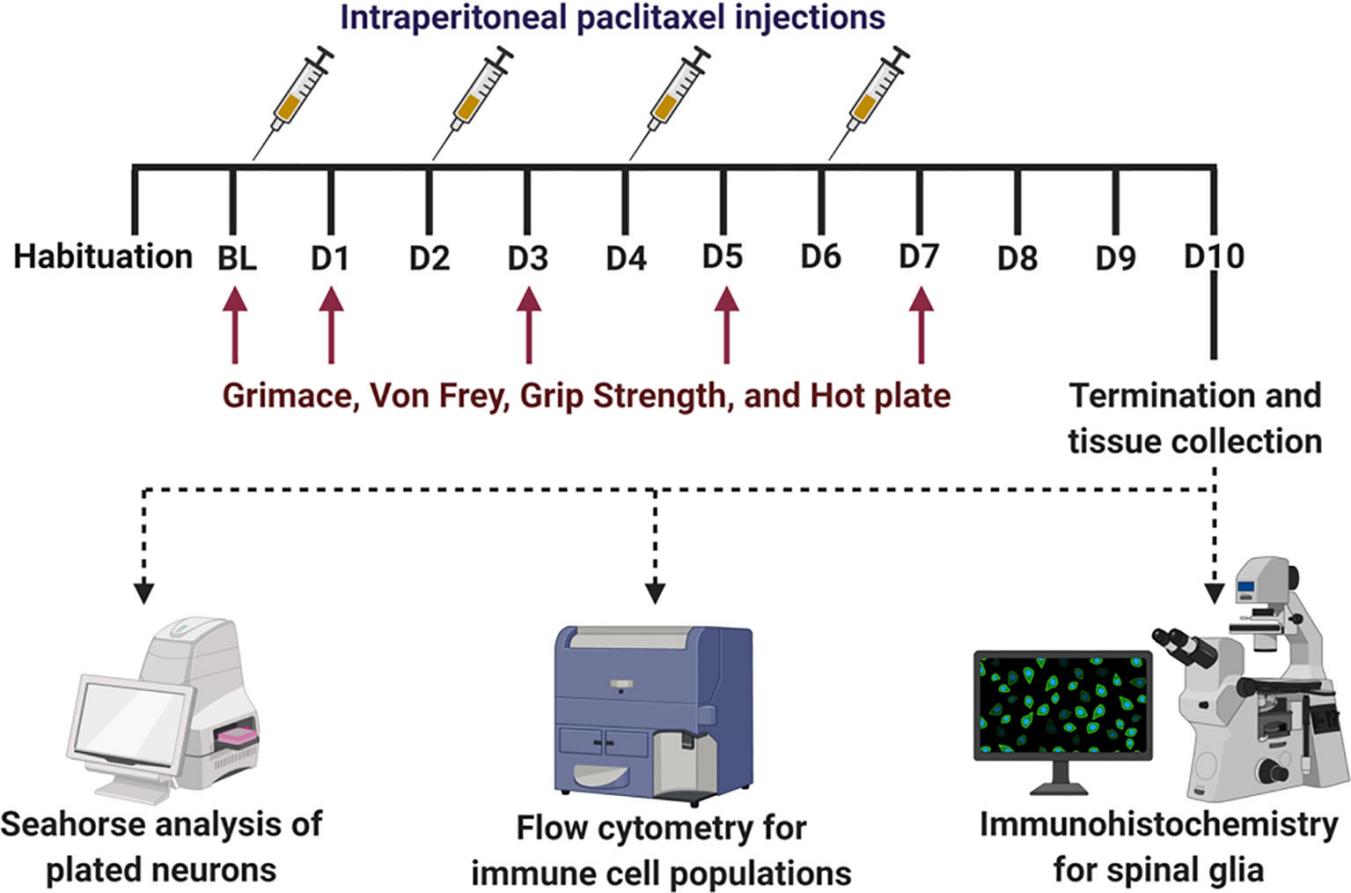
Experimental timeline with dosing regimen and terminal analysis. After habituation and baseline (BL) measures for behavior, all mice were subjected to intraperitoneal injection of paclitaxel (4 mg/kg per mouse) after BL i.e. on day 0, D2, D4, and D6 to induce peripheral neuropathy. Behavioral assays (mechanical hypersensitivity, grimace, grip strength, and thermal hypersensitivity) were measured on BL, D1, D3, D5, D7, and D9. On D10, animals were terminated and spinal cord, popliteal and inguinal lymph nodes, and DRG (L3 to T13) were collected. The lumbar portion of the spinal cord was used for immunohistochemical analyses for glia reactivity. The lymph nodes and DRG were used for flow cytometry analyses of immune cell populations. DRG neurons were plated and used for Seahorse analyses of bioenergetics.

Adult mice of both sexes (9-12 weeks old, 20-25g weight) were used for all experiments. eIF4E^S209A^ mice were generated on a C57BL/6 background in the Sonenberg laboratory at McGill University as previously described (41). These were a gift and further bred to maintain genotypes at the University of Texas at (UT) Dallas vivarium to generate our experimental cohorts. In-house animals were weaned between 21 and 28 days of age and tail-clipped to verify genotypes. Both wild-type (WT) and eIF4E^S209A^ gift mice were bred with breeder animals purchased from Jackson laboratory (WT). We used only homozygous eIF4E^S209A^ mutant animals and WT littermates for experiments. All animals were housed at the UT Dallas vivarium with standard temperature (20-25°C) and a 12-hour light/dark cycle (lights on from 6 AM to 6 PM). Mice were group housed in polypropylene ventilated cages with 4-5 animals per cage and provided *ad libitum* access to food and water. All behavioral experiments and data analysis were carried out from 9am to 12pm during the light cycle. All experiments were performed in accordance with protocols and standard operating procedures (SOPs) approved by the University of Texas at Dallas Institutional Animal Care and Use Committee (IACUC) and the Institution Biosafety and Chemical Safety Committee. The experimenters were blinded to animal genotypes and treatment groups for all assays. A timeline of experiments and dosing regimen is shown in **Figure 1**.

#### Induction of Chemotherapy-Induced Peripheral Neuropathy (CIPN) With Administration of Paclitaxel

Paclitaxel (European pharmacopoeia (EP) Reference standard purchased from Sigma, Y0000698) was dissolved in a vehicle (1:1 ratio of Kolliphor oil (Sigma, C5135) and 100% ethanol), to prepare stock paclitaxel solution (5mg/mL stored at 4°C). Working stock was freshly prepared on injection days by diluting stock solution with sterile 1× PBS. Paclitaxel injections were administered intraperitoneally every other day for four (4mg/kg/day) injections to induce peripheral neuropathy (16mg/kg total dose per animal, **Figure 1**) as previously described (44, 45), these studies show little to no differences of 2mg/kg versus 4mg/kg in the development of painful CIPN.

### Behavioral Experiments

Baseline readings for all behavior assays were taken twice, a day apart (days -1 and -3) before administration of the 1^st^ paclitaxel injection on day 0. Behavioral assessment for spontaneous pain using the grimace scale, mechanical hypersensitivity, grip strength, and thermal hyperalgesia were performed on days 1, 3, 5, 7, and 9, in that order. This was important to maintain non-confounding observations of evoked pain behavior using touch or heat stimuli after noting spontaneous pain. All behavior datasets are additionally represented as effect size. Effect size here is determined by calculating the cumulative difference between the value for each time point and the baseline value. All effect values were added to get effect size represented as an absolute number for each of the animal groups (46).

#### Assessment of Spontaneous Pain by Grimace Scale

Grimace is assessment of spontaneous pain using the observation of facial expression (47–49). Five parameters for facial grimace behavior were assessed: orbital tightening, ear position, cheek bulge, nose bulge, and whisker position. Each component was scored as follows: not present was given a score of “0”, moderately present was given a score of “1”, and obviously present was given a score of “2”. The score of the five components was averaged to give a mean grimace score (MGS).

#### Assessment of Mechanical Hypersensitivity by von Frey Filaments

Mechanical hypersensitivity was assessed using the previously described von Frey assay (50). Von Frey acrylic chamber (11cm long, 10 cm wide, and 4.5 cm height) with mesh floor, elevated 4.25 feet off the ground, were used for this assay. Animals were acclimated to the testing room and habituated to the von Frey apparatus and test environment for at least 1 hour prior to baseline and testing measurements. After 2 baseline recordings (one day rest in between the recordings), animals were randomly assigned to the different treatment groups. Mechanical hypersensitivity was determined by assessment of paw withdrawal threshold in response to the application of calibrated von Frey filaments (Stoelting, Illinois, USA) using the up-down method. A series of filaments with logarithmically incremental stiffness of 2.83, 3.22, 3.61, 3.84, 4.08, and 4.17 (converted to the 0.07, 0.16, 0.4, 0.6, 1, and 1.4 grams, respectively) were applied to the plantar surface of the hind paw and held for 2-3 seconds. A positive response was counted if there was a brisk withdrawal of the paw, paw shaking, and paw licking, or holding the paw in the air more than 2 seconds. The withdrawal threshold of both hind paws was measured and averaged.

#### Assessment of Muscle Dexterity by Grip Strength

Grip strength is a measure of functional pain in laboratory rodents and is measured with a grip strength test meter (IITC, California, USA). All animals were habituated to the apparatus for 5 minutes per animal, and were trained to grip the mesh wire surface prior to baseline or experiment. To measure, animals were gently placed on the rectangular wire meshed surface connected to the transducer in the grip strength meter and gently pulled away until they let go of the mesh. The maximal grip force was automatically measured by the transducer and recorded. Three trials were performed and averaged for both baseline measures and all experiments.

#### Assessment of Thermal Hyperalgesia by Hot Plate

Thermal sensitivity was assessed using the hot plate apparatus (IITC, California, USA). Animals were individually placed onto a surface maintained at 52°C, with their locomotion restricted by a Plexiglas chamber. Hind paw licking, shaking, and jumping was recorded as a positive response. Total time spent on the hot plate surface until a positive response was detected was measured and recorded as latency to response. The heating time cutoff was restricted to maximum 30 seconds to prevent tissue injury.

### Immunohistochemistry

Animals were anesthetized with intraperitoneal injection of Ketamine/Xylazine (100 mg/kg) and intracardially perfused with 1x PBS solution followed by 4% paraformaldehyde (PFA). Lumbar spinal cord tissue was collected, post fixed in 4% PFA for 4 hours at 4°C and cryoprotected in 30% sucrose for 48 hours at 4°C. Tissues were embedded in optimal cutting temperature (OCT) (Fisher Scientific; 23-730-571) followed by cryo-sectioning into 20 μm sections that were mounted on positively charged glass slides (VWR, 48311-703). Mounted sections were treated with 1M HCL for 30 minutes followed by neutralization with 0.1 M sodium borate (pH 8.5) for 10 minutes. The slides were washed with 1x PBS and pre-incubated with 5% normal goat serum in 0.2% Triton X-100 in 1x PBS solution to block nonspecific binding. Subsequently, sections were incubated with the primary antibodies overnight at 4°C (see **Table 1**). The next day, sections were incubated in respective secondary antibodies and treated with DAPI solution (1:5000 dilution, Sigma-Aldrich D9542). Stained sections were covered with Gelvatol mounting medium and cover slips (51) and images were taken on a Zeiss Axio-observer 7 microscope (Jena, Germany) for quantitative analysis. Representative images were taken on an Olympus FV3000RS confocal laser scanning microscope (Shinjuku, Tokyo, Japan). Images acquired for quantitative analysis were taken using identical exposure times and representative images at identical laser power. Image analysis was done using FIJI (ImageJ) and Cell Sens version 3.1 (Olympus, Japan) software.

**Table 1:** Antibodies used for IHC and flow cytometry.

Antibody	Company	Catalog number	Working dilution
*Antibodies used for IHC*			
Anti-Iba1	WAKO	019-19741	1:1000
Anti-GFAP	DAKO	Z-0331	1:1000
Anti-NeuN	EMD Millipore	MAB377	1:1000
Anti-ATF3	Abcam	ab207434	1:1000
Goat anti-mouse Alexa Fluor 488	Invitrogen	A21121	1:500
Goat anti-rabbit Alexa Fluor 647	Invitrogen	A21245	1:500
*Antibodies used for flow cytometry*			
Anti-CD16/32	eBioscience	16016185	1:2000
Anti-CD3 Alexa fluor 700 conjugate	eBioscience	56003280	1:200
Anti-CD4 Fluorescein isothiocyanate conjugate	eBioscience	11004185	1:200
Anti-CD8 Phycoerythrin conjugate	eBioscience	12-0081-83	1:200
Anti-CD25 eFluor 450 conjugate	eBioscience	48025182	1:200
Anti-CD44 eFluor780 conjugate	eBioscience	47044182	1:200
Anti-CCR7 Allophycocyanin conjugate	eBioscience	17197942	1:200
Anti-CD11b Allophycocyanin-Cy7 conjugate	Life Technologies	A15390	1:200
Anti-CD45 Brilliant violet 421 conjugate	Biolegend	103133	1:200
Anti-MHCII Alexa fluor 488 conjugate	eBioscience	11532282	1:2000
Anti-CD40 Phycoerythrin conjugate	eBioscience	120401-82	1:200

### Flow Cytometry

Flow cytometric analysis of isolated immune cells from lymph node and dissociated DRG was performed based on previous protocols with few modifications (52). In brief, lymph nodes (popliteal and inguinal) and DRG (lumbar and thoracic, T13 to L5) were collected in ice cold sterile DPBS (Hyclone, Logan, UT). Lymph nodes were passed through a 70 micron nylon mesh with flow buffer (0.5% bovine serum albumin with 0.02% glucose) while DRG were first digested with enzymes (details described in section 2.6) and then passed through 70 micron nylon mesh. Resultant cell suspensions were centrifuged at 400 × g for 6 minutes at 4 °C. Cell pellets were washed with cold 1x PBS and resuspended in pre-chilled flow buffer. Fc receptors were blocked by anti-CD16/CD32 purified antibody. Cells from lymph nodes were incubated with anti-CD3-Alexa Flour 700, anti-CD4-Fluorescein isothiocyanate, anti-CD8 Phycoerythrin, anti-CD25-eFluor 450, anti-CD44-eFluor780, and anti-CCR7-Allophycocyanin (see **Table 1** for antibody details). Cells from DRG were incubated with anti-CD11b-Allophycocyanin-Cy7, anti-CD45-Brillient violet 421, anti-MHCII-Alexa Fluor 488, and anti-CD40 Phycoerythrin antibodies. Appropriate compensation controls and isotypes were used for determination and gating. Cells were washed twice in ice cold flow buffer and resuspended in ice cold flow buffer. T_helper_ T-cells were initially identified with gating CD3 and CD4 cell surface markers, whereas cytotoxic T-cells were identified by gating for CD3 and CD8. These populations were further gated to identify activation and polarization (T_h1_ vs. T_h2_) with CD44 and CD25. CCR7 was also used to identify T-effector cells. Tissue activated macrophages were identified from CD45 separated cells with cell surface markers CD11b, MHC-II, and CD40. Stained samples were analyzed using a Becton-Dickinson Fortessa analyzer (Red Oaks, CA) and data were analyzed using FlowJo software (De Novo Software, Los Angeles, CA). For a complete list of antibodies used, please refer **Table 1**.

### Primary Dorsal Root Ganglia (DRG) Culture and Cellular Energetics

On day 10, animals were deeply anesthetized with isoflurane and decapitated. DRG were dissected bilaterally starting from T13 to L5 and transferred to pre-chilled 1x PBS containing 1% penicillin/streptomycin (ThermoFisher Scientific, 15070063). Samples were centrifuged at 400 x g for 4 minutes. Supernatants were removed and DRG were treated with Collagenase A (Sigma, 10103586001) and incubated in water bath at 37°C for 20 minutes, followed with treatment of Collagenase D (Sigma, 1188866001) for another 20 minutes. Cells were centrifuged at 400 x g and pellet was resuspended in Enzyme T (soybean trypsin inhibitor made up in 1 part bovine serum albumin and 1 part DMEM/F12 media) to stop the enzymatic reaction. Digested tissues were triturated approximately 30 times using a 1 ml pipette tip and passed through the 70 micron nylon mesh, with a subsequent wash with DMEM/F12 media (supplemented with 10% Fetal bovine serum and 1% penicillin/streptomycin). Resultant suspension was centrifuged at 400 x g for 5 minutes and resuspended in DMEM/F12 medium. The number of cells was counted using a hemocytometer with trypan blue dye exclusion. For bioenergetic profile, 70,000 cells were seeded directly in respective XFp Cell Culture Miniplates (Agilent, 103025-100) coated with poly-D-Lysine (Millipore Sigma, P0899). Next day, XF Assay media (Seahorse Bioscience) supplemented with 2 mM GlutaMAX, 5 mM glucose (Sigma), 1 mM sodium pyruvate (Fisher Scientific, Loughborough, United Kingdom), and pH adjusted to pH 7.4, was warmed to 37°C. Cells were incubated with this Seahorse media for 1 hour at 37°C, 0% CO2 incubator. The media was replaced with the Seahorse XF calibrant solution for another hour before running the Mito Stress according to manufacturer’s recommended protocol (53).

### Statistical Analysis

Data were analyzed using GraphPad Prism software (version 8.4) and expressed as mean ± standard error of the mean (SEM). For behavior, flow cytometry, and Seahorse datasets, two-way ANOVAs were performed followed by Tukey’s *post-hoc* for multiple comparisons. For Seahorse divided into phases of mitochondrial respiration, one-way ANOVA was performed with Sidak’s multiple comparison test. For IHC datasets, two-way ANOVA followed by Bonferroni’s *post-hoc* was performed for glia reactivity and Sidak’s *post-hoc* was used for ATF3. A *p* value of ≤ 0.05 was considered significant.

## Results

### Paclitaxel Induces Pain Like Behavior in Male and Female Mice, Which Is Mediated *via* eIF4E 

**Figure 2:**
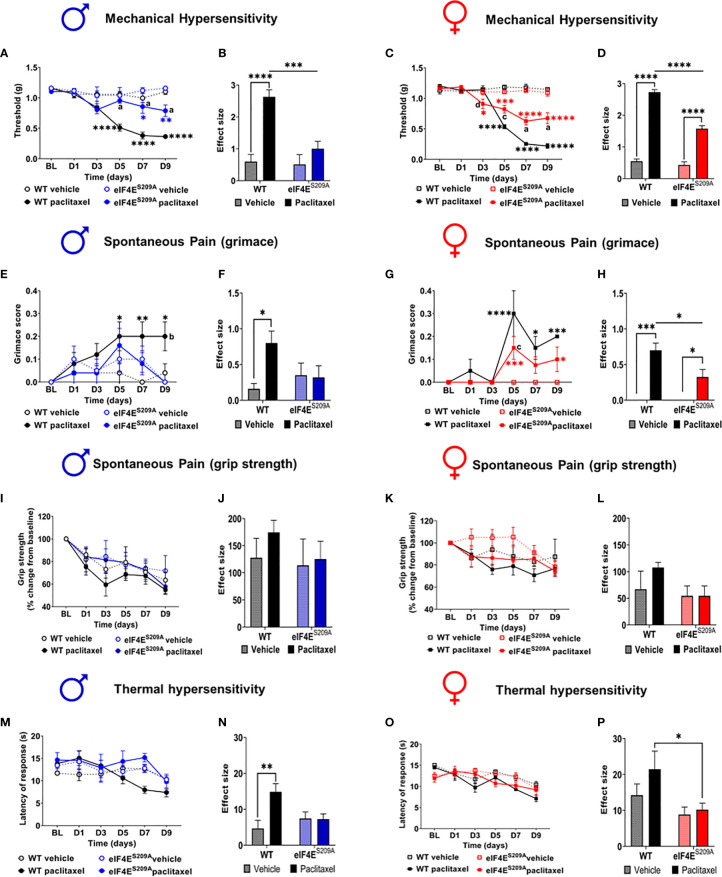
Paclitaxel induces pain like behavior in male and female mice, which is eIF4E dependent. **(A)** Mechanical hypersensitivity in male WT and eIF4E^S209A^ mice. Blue asterisks *p 0.0475, ** *p* 0.0014 for eIF4E vehicle VS paclitaxel, Black asterisks ****p<0.0001 for WT vehicle VS paclitaxel, a=*p*<0.0001 for WT paclitaxel VS eIF4E paclitaxel. **(B)** Effect size here is determined by calculating the cumulative difference between the value for each time point and the baseline value. Data for male mice shown as effect size, ****p<0.0001, ***p 0.0004. **(C)** Mechanical hypersensitivity in female WT and eIF4E^S209A^ mice. Red asterisks *p 0.0261 , ***p 0.003 for eIF4E vehicle VS paclitaxel, Black asterisks ****p<0.0001 for WT vehicle VS paclitaxel, a=*p*<0.0001, c=*p* 0.0029, d=*p* 0.0286 for WT paclitaxel VS eIF4E paclitaxel. **(D)** Data for female mice shown as effect size, ****p<0.0001. **(E)** Spontaneous pain assessment by grimace score in male mice. *p 0.0495, **p 0.008 for WT vehicle VS paclitaxel. **(F)** Grimace scores shown as effect size for male mice, *p 0.0302. **(G)** Grimace scores for female mice. *p 0.0153 for D7 WT vehicle VS paclitaxel, 0.0328 for D9 for eIF4E vehicle VS paclitaxel, ***p 0.0004 for D5 for eIF4E vehicle VS paclitaxel, 0.0009 for D9 for WT vehicle VS paclitaxel, ****p<0.0001 for WT vehicle VS paclitaxel. **(H)** Grimace scores shown as effect size for female mice, *p 0.0249 compared for treatment, *p 0.0293 compared for genotype, ***p 0.0005. **(I)** Grip strength for male mice. **(J)** Grip strength for males shown as effect size. **(K)** Grip strength for female mice. **(L)** Grip strength in females shown as effect size. **(M)** Thermal hypersensitivity in male mice. **(N)** Thermal hypersensitivity in males shown as effect size. **p 0.0072. **(O)** Thermal hypersensitivity in female mice. **(P)** Thermal hypersensitivity in females shown as effect size, *p 0.0453. All data are presented as mean ± standard error of the mean, males: n = 6 mice per group; females: n = 12 mice per group. Two-way ANOVA was performed with Tukey’s *post-*hoc for multiple comparisons. For all line graphs, asterisks indicate significant differences between vehicle VS paclitaxel-treated for each genotype at indicated time points. “a” (*p* < 0.0001), “b” (*p* < 0.001), “c” (*p* < 0.01), and “d” (*p* < 0.05) depict significant difference between paclitaxel-treated WT and eIF4E^S209A^ mice at indicated time points.

Previously, it has been shown that an intraperitoneal injection regimen of paclitaxel leads to development of long-lasting mechanical hypersensitivity in WT male and female mice during later stages of CIPN (45, 54). We investigated whether cap-dependent translation was involved in earlier stages of CIPN development after intraperitoneal paclitaxel administration. We found a significant paclitaxel-dependent increase in mechanical hypersensitivity in WT mice of both sexes, compared to vehicle treatment (**Figures 2A–D** and **Table 2**). In the absence of eIF4E phosphorylation, this increase was observed only in females (**Figures 2C, D** and **Table 2**), not male mice (**Figures 2A, B** and **Table 2**). Compared to WT paclitaxel-treated mice, the eIF4E^S209A^ paclitaxel-treated mice had significantly lower mechanical hypersensitivity (**Figures 2B, D** and **Table 2**) in both sexes. These findings indicate that eIF4E phosphorylation plays a protective role in the development of mechanical hypersensitivity after paclitaxel administration, but this protection is to a lesser extent in females. We assessed spontaneous pain by grimace and grip strength. Paclitaxel treatment induced more grimacing in male mice in an eIF4E-dependent manner (**Figures 2E, F** and **Table 2**). For females, spontaneous pain was higher with paclitaxel administration, but eIF4E^S209A^ females had lower grimacing compared to WT (**Figures 2G, H**). This indicates that eIF4E affects pain perception associated with paclitaxel-induced peripheral neuropathy. There were no changes in the grip strength for either sex, genotype, or treatment (**Figures 2I–L**). Male WT mice showed the maximum latency of response to hot plate with paclitaxel treatment, with no difference in eIF4E^S209A^ male mice (**Figures 2M** and **Table 2**), indicating that eIF4E is required for mediating response to thermal stimuli after paclitaxel. On the other hand, females of both genotypes did not show significant changes in latency with paclitaxel treatment compared to vehicle (**Figures 2O, P** and **Table 2**). Interestingly, eIF4E^S209A^ females treated with paclitaxel had a significantly reduced latency of response to heat stimuli compared to their WT counterparts (**Figure 2P** and **Table 2**). Taken together, our data suggests that eIF4E is important for mechanisms involved in CIPN development early in both sexes.

**Table 2:** Statistical values for behavior data analysis.

Dataset	Main effect		Interactions		Multiple comparisons		
	F (DFn,DFd)	*p*-value	F (DFn,DFd)	*p*-value	Effect	Groups	*p*-value
Mechanical hypersensitivity male mice	Treatment: F (1, 25) = 12.26	**0.0018**	F (1, 25) = 9.797	**0.0044**	Treatment	Vehicle	0.9938
						Paclitaxel	**0.0004**
	Genotype: F (1, 25) = 26.48	**<0.0001**			Genotype	WT	**<0.0001**
						eIF4E^S209A^	0.5380
Mechanical hypersensitivity female mice	Treatment: F (1, 19) = 251.1	**<0.0001**	F (1, 19) = 24.49	**<0.0001**	Treatment	Vehicle	0.8600
						Paclitaxel	**<0.0001**
	Genotype: F (1, 19) = 36.87	**<0.0001**			Genotype	WT	**<0.0001**
						eIF4E^S209A^	**<0.0001**
Grimace male mice	Treatment: F (1, 15) = 4.236	**0.0574**	F (1, 15) = 5.111	**0.0391**	Treatment	Vehicle	0.8146
						Paclitaxel	0.1282
	Genotype: F (1, 15) = 0.9575	0.3433			Genotype	WT	**0.0302**
						eIF4E^S209A^	0.9990
Grimace female mice	Treatment: F (1, 19) = 34.43	**<0.0001**	F (1, 19) = 4.609	**0.0449**	Treatment	Vehicle	>0.9999
						Paclitaxel	**0.0293**
	Genotype: F (1, 19) = 4.609	**0.0449**			Genotype	WT	**0.0005**
						eIF4E^S209A^	**0.0249**
Grip strength male mice	Treatment: F (1, 18) = 0.6734	0.4226	F (1, 18) = 0.2471	0.6251	N/A		
	Genotype: F (1, 18) = 0.7916	0.3854					
Grip strength female mice	Treatment: F (1, 16) = 0.8995	0.3570	F (1, 16) = 0.8995	0.3570	N/A		
	Genotype: F (1, 16) = 2.349	0.1449					
Thermal hypersensitivity male mice	Treatment: F (1, 24) = 6.294	**0.0193**	F (1, 24) = 6.743	**0.0158**	Treatment	Vehicle	0.7602
						Paclitaxel	**0.0579**
	Genotype: F (1, 24) = 1.455	0.2395			Genotype	WT	**0.0072**
						eIF4E^S209A^	>0.9999
Thermal hypersensitivity female mice	Treatment: F (1, 19) = 2.332	0.1432	F (1, 19) = 1.090	0.3097	Treatment	Vehicle	0.5527
						Paclitaxel	**0.0453**
	Genotype: F (1, 19) = 8.729	**0.0081**			Genotype	WT	0.4059
						eIF4E^S209A^	0.9762

Two-way ANOVA was performed followed by Tukey’s post-hoc for multiple comparisons between genotypes and treatments for each sex. p ≤ 0.05 was considered significant (bolded).

### Paclitaxel Dysregulates CD4^+^ and CD8^+^ T-Cell Subpopulations in an eIF4E-Dependent Manner

**Figure 3:**
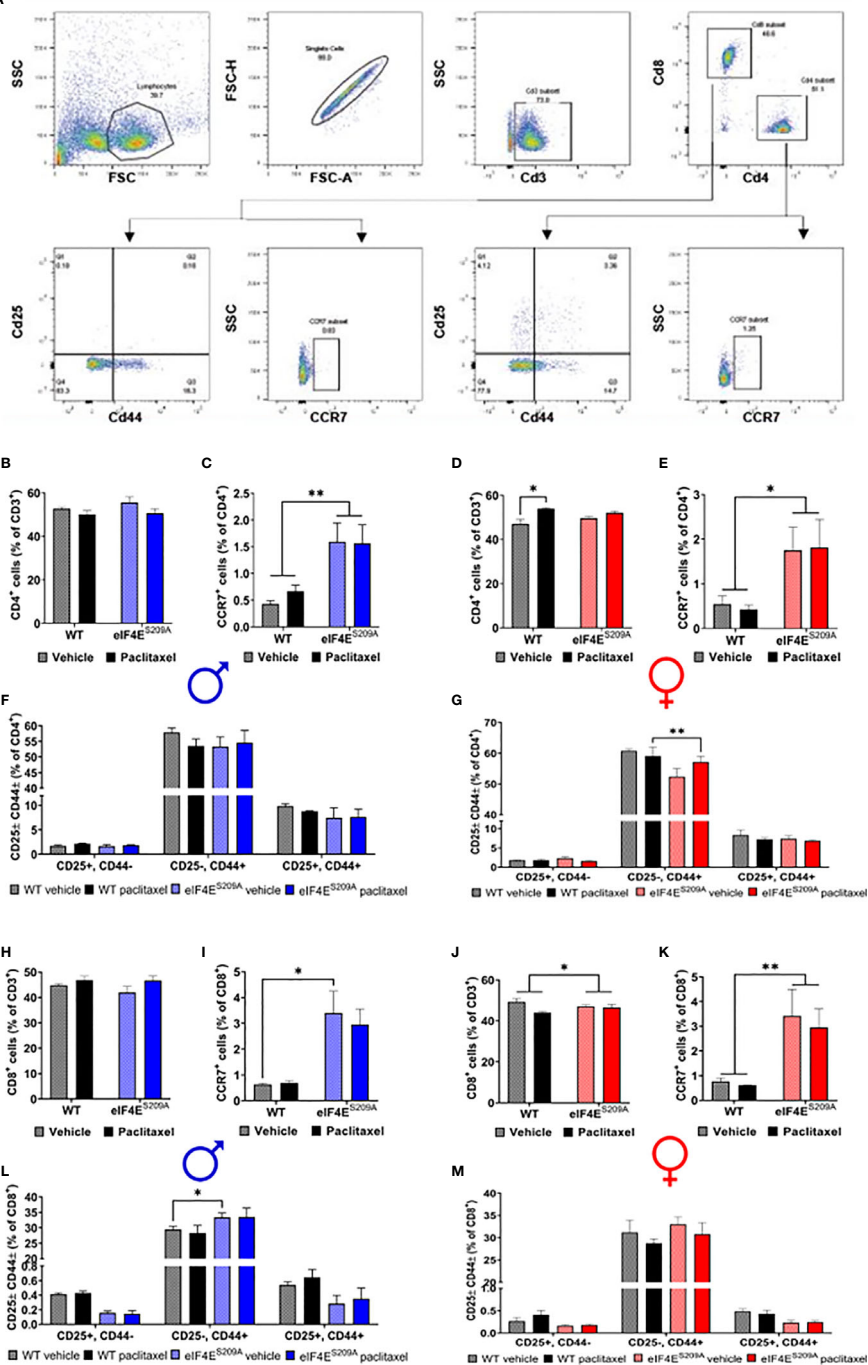
Paclitaxel dysregulates CD4^+^ and CD8^+^ T-cell subpopulations in an eIF4E-dependent manner. **(A)** Gating strategy used for flow cytometry of lymphocytes from popliteal and inguinal lymph nodes. T-cells were separated from the whole lymphoid cells population using CD3. CD3^+^ cells were then gated for CD4^+^ T-cell and CD8^+^ T-cells. For each of these subsets, cells were further gated for CCR7 or CD25 and CD44. **(B)** Quantification of CD4^+^ T-cells from the CD3 population from male mice. **(C)** CCR7^+^ T-cells from isolated CD4^+^ population in males, **p 0.0063. **(D)** CD4^+^ cells gated for CD25 and/or CD44 in males. **(E)** Quantification of CD4^+^ T-cells from the CD3^+^ population from female mice, *p 0.0101. **(F)** CCR7^+^ T-cells from isolated CD4^+^ population in female mice, *p 0.0250. **(G)** CD4^+^ cells gated for CD25 and/or CD44 in female mice, **p 0.0011. **(H)** Quantification of CD8^+^ T-cells from the CD3^+^ population from male mice. **(I)** CCR7^+^ T-cells from isolated CD8^+^ population in males, *p 0.0433. **(J)** CD8^+^ cells gated for CD25 and/or CD44 in males, *p 0.0396. **(K)** Quantification of CD8^+^ T-cells from the CD3 population from female mice, *p 0.041. **(L)** CCR7^+^ T-cells from isolated CD8^+^ population in female mice, **p 0.0097. **(M)** CD8^+^ cells gated for CD25 and/or CD44 in female mice. All data are presented as mean ± standard error of the mean, n = 3 mice each for male/female WT vehicle-treated and WT paclitaxel groups, n = 4 mice each for male/female eIF4E^S209A^ vehicle-treated and paclitaxel. Two-way ANOVA was performed with Tukey’s *post-*hoc for multiple comparisons.

An obvious early contribution of adaptive immune cells in the development of paclitaxel-induced peripheral neuropathy has not been shown till date. Given that the paclitaxel was administered peripherally, T-cells are activated in the lymph nodes, and neuropathy develops in the limbs, we chose to study subpopulations of T-cells from the draining (popliteal and inguinal) lymph nodes that are CD4^+^ (T_helper_) CD8^+^, CD25^+/-^ and CD44^+/-^ (activated T-cells), CCR7^+^ (effector T-cells) (**Figures 3A–F**). We found a significant increase in CD4^+^ T-cells in WT female mice injected with paclitaxel, but not males (**Figures 3A, B, E** and **Table 3**). The CD4^+^ subset was further analyzed for CCR7, the marker for lymphocyte homing to lymph nodes as well as T-cell priming (55, 56). The CD4+CD25-CD44+ subset was significantly reduced in eIF4E^S209A^ females treated with paclitaxel compared to WT (**Figure 3G**, **Table 3**). The CD4^+^CCR7^+^ subpopulation was significantly higher in eIF4E^S209A^ mice of both sexes independent of paclitaxel treatment (**Figures 3C, F** and **Table 3**). This suggests that inhibiting cap-dependent translation upregulates T-cell priming and activation of T cells. We found significant downregulation of CD8^+^ T-cells in female eIF4E^S209A^ mice compared to WT, irrespective of treatment (**Figures 3G–L**, **Table 3**). This population was unchanged in male mice (**Figure 3H**). CD8^+^CCR7^+^ T-cells, i.e. activated memory T-cells were higher in eIF4E^S209A^ mice (**Figures 3I, L** and **Table 3**) regardless of sex or treatment. CD8^+^CD44^+^CD25^-^ T-cells were increased in paclitaxel-treated eIF4E^S209A^ male mice compared to WT males treated with paclitaxel (**Figure 3J** and **Table 3**). There were no differences in female mice for this subpopulation (**Figure 3M** and **Table 3**). Overall, our data shows that paclitaxel induces activation and/or expansion of T-helper, memory T, and effector T-cells, which is modulated in an eIF4E-dependent manner.

**Table 3:** Statistical values for flow cytometry data analysis.

Dataset	Main effect		Interactions		Multiple comparisons		
	F (DFn,DFd)	*p*-value	F (DFn,DFd)	*p*-value	Effect	Groups	*p*-value
*Flow cytometry data analysis for T-cells from popliteal and inguinal lymph nodes*							
CD4^+^ males	Treatment: F (1, 10) = 3.139	0.1069	F (1, 10) = 0.3061	0.5922	N/A		
	Genotype: F (1, 10) = 0.6463	0.4401					
CD4^+^CCR7^+^ males	Treatment: F (1, 10) = 0.1196	0.7367	F (1, 10) = 0.1991	0.6650	Treatment	Vehicle	0.0814
						Paclitaxel	0.2116
	Genotype: F (1, 10) = 11.86	**0.0063**			Genotype	WT	0.9514
						eIF4E^S209A^	0.9998
CD4^+^CD44^±^CD25^±^ males	Cell populations: F (2, 30) = 809.1	**<0.0001**	F (6, 30) = 0.2916	0.9362	No genotype or treatment difference within CD4^+^CD44^+^CD25^-^, CD4^+^CD44^-^CD25^+^, or CD4^+^CD44^+^CD25^+^ populations.		
	Genotype-treatment: F (3, 30) = 0.6686	0.5780					
CD4^+^ females	Treatment: F (1, 10) = 17.44	**0.0019**	F (1, 10) = 3.920	0.0759	Treatment	Vehicle	0.3920
						Paclitaxel	0.6742
	Genotype: F (1, 10) = 0.1340	0.7219			Genotype	WT	**0.0101**
						eIF4E^S209A^	0.3831
CD4^+^CCR7^+^ females	Treatment: F (1, 10) = 0.0027	0.9591	F (1, 10) = 0.0341	0.8571	Treatment	Vehicle	0.3583
						Paclitaxel	0.2532
	Genotype: F (1, 10) = 6.933	**0.0250**			Genotype	WT	0.9985
						eIF4E^S209A^	0.9996
CD4^+^CD44^±^CD25^±^ females	Cell populations: F (2, 30) = 1893	**<0.0001**	F (6, 30) = 2.406	0.0512	CD4^+^CD44^+^CD25^-^ Genotype ns		
	Genotype-treatment: F (3, 30) = 2.452	0.0827			CD4^+^CD44^+^CD25^-^ Treatment	Vehicle	**0.0011**
						Paclitaxel	0.7767
CD8^+^ males	Treatment: F (1, 10) = 3.180	0.1049	F (1, 10) = 0.5157	0.4891	N/A		
	Genotype: F (1, 10) = 0.6004	0.4564					
CD8^+^CCR7^+^ males	Treatment: F (1, 10) = 0.0917	0.7681	F (1, 10) = 0.1555	0.7016	Treatment	Vehicle	**0.0433**
						Paclitaxel	0.1044
	Genotype: F (1, 10) = 16.48	**0.0023**			Genotype	WT	>0.9999
						eIF4E^S209A^	0.9491
CD8^+^CD44^±^CD25^±^ males	Cell populations: F (2, 30) = 772.5	**<0.0001**	F (6, 30) = 1.591	0.1842	CD8^+^CD44^+^CD25^-^ Genotype ns		
	Genotype-treatment: F (3, 30) = 1.096	0.3660			CD8^+^CD44^+^CD25^-^Treatment	Vehicle	0.1579
						Paclitaxel	**0.0396**
CD8^+^ females	Treatment: F (1, 10) = 5.496	**0.0410**	F (1, 10) = 3.684	0.0839	Treatment	Vehicle	0.6174
						Paclitaxel	0.4863
	Genotype: F (1, 10) = 0.0273	0.8720			Genotype	WT	0.0730
						eIF4E^S209A^	0.9875
CD8^+^CCR7^±^ females	Treatment: F (1, 10) = 0.1574	0.6999	F (1, 10) = 0.0371	0.8510	Treatment	Vehicle	0.1422
						Paclitaxel	0.2125
	Genotype: F (1, 10) = 10.15	**0.0097**			Genotype	WT	0.9991
						eIF4E^S209A^	0.9681
CD8^+^CD44^±^CD25^±^ females	Cell populations: F (2, 30) = 788.2	**<0.0001**	F (6, 30) = 0.7166	0.6392	No genotype or treatment difference within CD8^+^CD44^+^CD25^-^, CD8^+^CD44^-^CD25^+^, or CD8^+^CD44^+^CD25^+^ populations.		
	Genotype-treatment: F (3, 30) = 0.5383	0.6597					
*Flow cytometry data analysis for myeloid cells from DRG*							
Males CD11b^+^CD45^+^CD40^±^MHCII^±^	Cell populations: F (2, 24) = 84.59	**<0.0001**	F (6, 24) = 1.803	0.1410	CD40^+^MHCII^+^ Treatment ns		
	Genotype-treatment: F (3, 24) = 1.139	0.3535			CD40^+^MHCII^+^ Genotype	WT	0.4651
						eIF4E^S209A^	**0.0275**
Females CD11b^+^CD45^+^CD40^±^MHCII^±^	Cell populations: F (2, 24) = 141.3	**<0.0001**	F (6, 24) = 4.720	**0.0026**	CD40^+^MHCII^+^ Treatment	Vehicle	**0.0140**
						Paclitaxel	**0.0055**
	Genotype-treatment: F (3, 24) = 5.042	**0.0075**			CD40^+^MHCII^+^ Genotype	WT	0.5182
						eIF4E^S209A^	**<0.0001**

Two-way ANOVA was performed followed by Tukey’s post-hoc for multiple comparisons between genotypes and treatments for each sex. p ≤ 0.05 was considered significant (bolded).

### Paclitaxel Induces Increased Infiltration of Activated Myeloid Cells in DRG in Absence of eIF4E Phosphorylation

**Figure 4:**
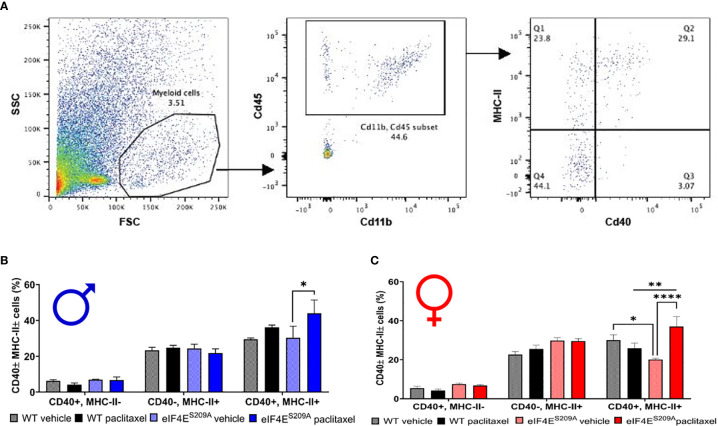
Paclitaxel induces activation of antigen-presenting myeloid cells in eIF4E^S209A^ mice DRG. **(A)** Gating strategy for isolating myeloid APCs from mouse DRG. Total cells were gated for CD11b and CD45 to separate out immune cells. The CD11b^+^ CD45^+^ subset was then gated for MHC-II^+^ and/or CD40^+^ cells. **(B)** Quantification of CD40^+^ and/or MHC-II^+^ cells from male mice, *p 0.0275. **(C)** Quantification of CD40^+^ and/or MHC-II^+^ cells from female mice, *p 0.014, **p 0.0055, ****p<0.0001. All data are presented as mean ± standard error of the mean, n = 3 mice each for male/female WT vehicle-treated and WT paclitaxel groups, n = 4 mice each for male/female eIF4E^S209A^ vehicle-treated and paclitaxel. Two-way ANOVA was performed with Tukey’s *post-*hoc for multiple comparisons.

We further investigated if peripheral neuropathy leads to the recruitment of macrophages into the DRG. No studies to date have shown the recruitment of the macrophages and the activation state of macrophages in DRG after CIPN. We looked for the infiltration and activation of macrophages using the CD11b^+^-CD45^+^ and MHCII^+^-CD40^+^ cells respectively in male and female mice. We identified macrophages within DRG by gating for CD11b^+^ and CD45^+^ cells. This population was further gated for MHCII^+^ and CD40^+^ to assess activated macrophage subsets (**Figure 4A**). We found that CD11b^+^-CD45^+^-CD40^+^-MHC-II^-^ and CD11b^+^-CD45^+^-CD40^-^-MHC-II^+^ populations were unchanged in both sexes with paclitaxel or lack of eIF4E (**Figures 4B, C** and **Table 3**), indicative of no effect on dendritic cells or tissue-resident macrophages. We found a significant increase in CD11b^+^-CD45^+^-CD40^+^-MHC-II^+^ cells in male and female eIF4E^S209A^ mice treated with paclitaxel compared to vehicle. Additionally, in female mice, this subpopulation was significantly higher in paclitaxel-treated eIF4E^S209A^ animals compared to paclitaxel-treated WT (**Figure 4C** and **Table 3**). This indicates that antigen-presenting cells (APCs) are upregulated with paclitaxel treatment in absence of eIF4E. Interestingly, female eIF4E^S209A^ vehicle-treated mice had a significantly lower proportion of activated APCs compared to WT (**Table 3**). This shows that cap-dependent translation may be facilitating the recruitment of activated myeloid cells as a result of paclitaxel treatment, which in turn could be activating or expanding the T-cell populations.

### Paclitaxel Alters Mitochondrial Function of DRG Neurons in an eIF4E-Dependent Manner 

**Figure 5:**
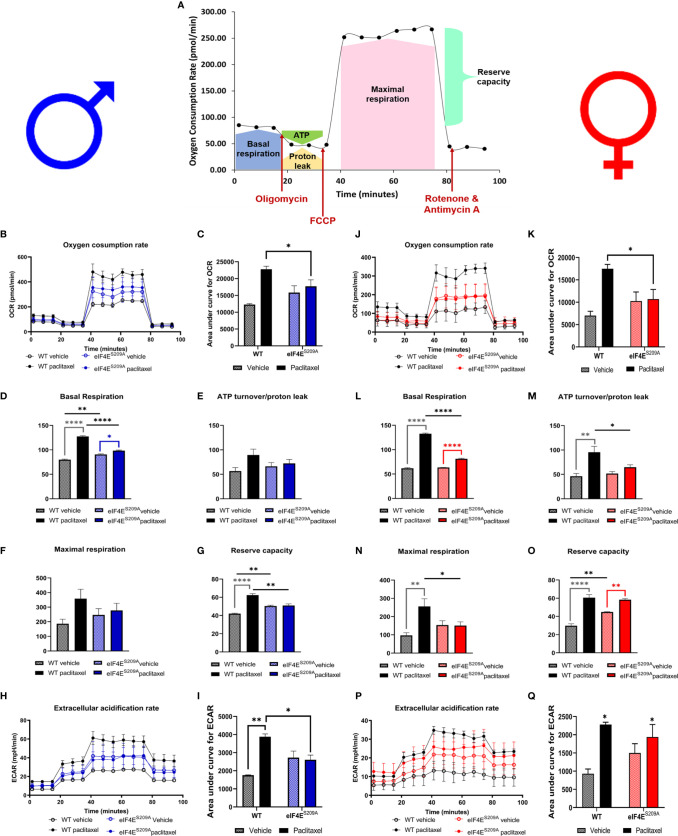
Paclitaxel alters mitochondrial function of DRG neurons in an eIF4E-dependent manner. Dissociated dorsal root ganglion (DRG) neurons from WT and eIF4E^S209A^ male and female mice were analyzed by the Seahorse Mito Stress test. **(A)** Graphic showing different phases of mitochondrial respiration. Adapted from the Agilent Seahorse XFp Cell Mito Test Kit User Guide published by Agilent Technologies. **(B)** Oxygen consumption rate (OCR) through phases of the Mito Stress test for male mice. **(C)** OCR data in male mice shown as area under the curve (AUC), *p 0.0136. **(D-G)** OCR data for male mice broken down into phases of mitochondrial respiration. Basal respiration: *p 0.0134, **p 0.0018, ****p<0.0001. Reserve capacity: **p 0.0063 for WT vs eIF4E^S209A^ vehicle, **p 0.001 for WT vs eIF4E^S209A^ paclitaxel. **(H)** Extracellular acidification rate (ECAR) for male mice. **(I)** ECAR data in male mice shown as AUC, *p 0.0303 for WT vs eIF4E^S209A^ vehicle, **p 0.0021 for WT vehicle vs WT paclitaxel groups. **(J)** OCR through phases of the Mito Stress test for female mice. **(K)** OCR data in female mice shown as AUC, *p 0.0455. **(L–O)** OCR data for female mice broken down into phases of mitochondrial respiration. Basal respiration: ****p<0.0001, ATP turnover: *p 0.0481, **p 0.0021, maximal respiration: *p 0.0417, **p 0.0011, reserve capacity: **p 0.0037 for WT vs eIF4E^S209A^ vehicle, **p 0.074 for eIF4E^S209A^ vehicle vs eIF4E^S209A^ paclitaxel, ****p<0.0001. **(P)** ECAR for female mice. **(Q)** ECAR data in female mice shown as AUC, *p 0.0116 for both genotypes vehicle vs paclitaxel-treated groups. All data are presented as mean ± standard error of the mean, n = 3-4 mice each for male/female WT vehicle-treated and WT paclitaxel groups, n = 4-7 mice each for male/female eIF4E^S209A^ vehicle-treated and paclitaxel. For analyzing AUC data, two-way ANOVA was performed with Tukey’s *post-*hoc for multiple comparisons. For analyzing each phase of mitochondrial respiration, an ordinary one-way ANOVA was used with Sidak’s *post-hoc* for multiple comparisons.

We investigated mitochondrial function in DRG neurons to examine if paclitaxel changed the bioenergetic profiles (**Figures 5A–Q** and **Table 4**) in an eIF4E-dependent manner. The overall oxygen consumption rate (OCR) was lower in paclitaxel-treated eIF4E^S209A^ males and females compared to paclitaxel-treated WT (**Figures 5B, C, J, K** and **Table 4**) whereas the overall extracellular acidification rate (ECAR) was higher with paclitaxel treatment in both sexes and lower in paclitaxel-treated eIF4E^S209A^ males compared to paclitaxel-treated WT males (**Figures 5H, I, P, Q** and **Table 4**). This suggests that lack of eIF4E causes a shift in mitochondrial respiration with paclitaxel treatment. We divided the different phases of the OCR bioenergetic profile into basal respiration (before oligomycin treatment), ATP production (after oligomycin treatment and before FCCP – 2-[2-[4-(trifluoromethoxy)phenyl]hydrazinylidene]-propanedinitrile treatment), maximal respiration (after FCCP and before antimycin A and rotenone), and non-mitochondrial respiration or reserve capacity (**Figure 5A**). For males, we found a significant increase in the basal respiration and non-mitochondrial respiration after paclitaxel treatment in WT, but not eIF4E^S209A^ mice (**Figures 5D, G** and **Table 4**), but no change in ATP turnover or maximal respiration (**Figures 5E, F** and **Table 4**). For female mice, we found that basal respiration and non-mitochondrial respiration was increased after paclitaxel treatment for both genotypes (**Figures 5L, O** and **Table 4**). ATP turnover and maximal respiration was also higher in WT females treated with paclitaxel compared to vehicle and in eIF4E^S209A^ females compared to paclitaxel-treated WT (**Figures 5M, N** and **Table 4**). Taken together, our data shows that sensory neurons in the DRG undergo an excited mitochondrial shift with paclitaxel treatment in an eIF4E-dependent manner in both sexes. Paclitaxel increased all aspects of mitochondrial respiration in females but did not affect ATP turnover and maximal respiration in males, which indicates divergent effects of paclitaxel in eIF4E absence in both sexes.

**Table 4:** Statistical values for DRG neuron mitochondrial respiration.

Dataset	Main effect		Interaction		Multiple comparisons			
	F (DFn,DFd)	p-value	F (DFn,DFd)	p-value	Effect		Groups	p-value
Males OCR AUC	Treatment: F (1, 11) = 11.85	**0.0055**	F (1, 11) = 5.817	**0.0345**	Treatment		Vehicle	0.5347
							Paclitaxel	0.2303
	Genotype: F (1, 11) = 0.1714	0.6868			Genotype		WT	**0.0136**
							eIF4E^S209A^	0.8475
Females OCR AUC	Treatment: F (1, 17) = 6.074	**0.0247**	F (1, 17) = 5.158	**0.0364**	Treatment		Vehicle	0.7519
							Paclitaxel	0.1426
	Genotype: F (1, 17) = 0.6271	0.4393			Genotype		WT	**0.0455**
							eIF4E^S209A^	0.9982
Males ECAR AUC	Treatment: F (1, 11) = 13.04	**0.0041**	F (1, 11) = 16.26	**0.0020**	Treatment		Vehicle	0.1313
							Paclitaxel	**0.0303**
	Genotype: F (1, 11) = 0.2927	0.5993			Genotype		WT	**0.0021**
							eIF4E^S209A^	0.9866
Females ECAR AUC	Treatment: F (1, 17) = 7.989	**0.0116**	F (1, 17) = 2.068	0.1686	Treatment		Vehicle	0.6269
							Paclitaxel	0.8523
	Genotype: F (1, 17) = 0.1287	0.7242			Genotype		WT	0.0792
							eIF4E^S209A^	0.6287
**Breakdown datasets for OCR**	**Interaction**	**p-value**	**Multiple comparisons - groups compared**					**p-value**
Males basal respiration	F (3, 8) = 233.2	**<0.0001**	WT vehicle		WT paclitaxel			**<0.0001**
			eIF4E^S209A^ vehicle		eIF4E^S209A^ paclitaxel			**0.0134**
			WT vehicle		eIF4E^S209A^ vehicle			**0.0018**
			WT paclitaxel		eIF4E^S209A^ paclitaxel			**<0.0001**
Females basal respiration	F (3, 8) = 838.7	**<0.0001**	WT vehicle		WT paclitaxel			**<0.0001**
			eIF4E^S209A^ vehicle		eIF4E^S209A^ paclitaxel			**<0.0001**
			WT vehicle		eIF4E^S209A^ vehicle			0.8413
			WT paclitaxel		eIF4E^S209A^ paclitaxel			**<0.0001**
Males ATP turnover	F (3, 12) = 2.384	0.1203	WT vehicle		WT paclitaxel			0.0905
			eIF4E^S209A^ vehicle		eIF4E^S209A^ paclitaxel			0.9832
			WT vehicle		eIF4E^S209A^ vehicle			0.9188
			WT paclitaxel		eIF4E^S209A^ paclitaxel			0.5864
Females ATP turnover	F (3, 12) = 8.828	**0.0023**	WT vehicle		WT paclitaxel			0.0021
			eIF4E^S209A^ vehicle		eIF4E^S209A^ paclitaxel			0.6736
			WT vehicle		eIF4E^S209A^ vehicle			0.9766
			WT paclitaxel		eIF4E^S209A^ paclitaxel			0.0481
Males maximal respiration	F (3, 28) = 2.233	0.1064	WT vehicle		WT paclitaxel			0.0661
			eIF4E^S209A^ vehicle		eIF4E^S209A^ paclitaxel			0.9857
			WT vehicle		eIF4E^S209A^ vehicle			0.8539
			WT paclitaxel		eIF4E^S209A^ paclitaxel			0.6683
Females maximal respiration	F (3, 28) = 5.986	**0.0028**	WT vehicle		WT paclitaxel			**0.0011**
			eIF4E^S209A^ vehicle		eIF4E^S209A^ paclitaxel			>0.9999
			WT vehicle		eIF4E^S209A^ vehicle			0.4836
			WT paclitaxel		eIF4E^S209A^ paclitaxel			**0.0417**
Males non-mitochondrial respiration (reserve capacity)	F (3, 8) = 42.18	**<0.0001**	WT vehicle		WT paclitaxel			**<0.0001**
			eIF4E^S209A^ vehicle		eIF4E^S209A^ paclitaxel			0.9978
			WT vehicle		eIF4E^S209A^ vehicle			**0.0063**
			WT paclitaxel		eIF4E^S209A^ paclitaxel			**0.0010**
Females non-mitochondrial respiration (reserve capacity)	F (3, 8) = 46.31	**<0.0001**	WT vehicle		WT paclitaxel			**<0.0001**
			eIF4E^S209A^ vehicle		eIF4E^S209A^ paclitaxel			**0.0074**
			WT vehicle		eIF4E^S209A^ vehicle			**0.0037**
			WT paclitaxel		eIF4E^S209A^ paclitaxel			0.9212

Two-way ANOVA was performed on AUC values for OCR and ECAR followed by Tukey’s post-hoc for multiple comparisons between genotypes and treatments for each sex. For breakdown data of mitochondrial respiration, ordinary one-way ANOVAs were used to analyze mean values of four groups (WT vehicle, WT paclitaxel, eIF4E^S209A^ vehicle, and eIF4ES^209A^ paclitaxel), followed by Sidak’s post-hoc for multiple comparisons between genotypes and treatments for each sex. p ≤ 0.05 was considered significant (bolded). OCR, oxygen consumption rate; ECAR, extracellular acidification rate.

### Sensory Neuron Damage by Paclitaxel Is Similar for Both Sexes and Genotypes

**Figure 6:**
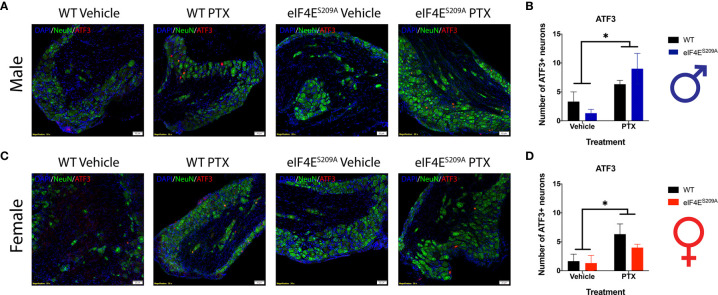
ATF3+ neurons are observed in DRG of all animals subjected to Paclitaxel. **(A, B**) Representative composite images showing ATF3^+^ cells (red) and NeuN^+^ cells (green) for male mice and their quantitation, respectively. *p 0.0114 for males’ vehicle vs paclitaxel groups. **(C, D)** Representative composite images showing ATF3^+^ cells (red) and NeuN^+^ cells (green) for female mice and their quantitation, respectively. *p 0.0218 for females for vehicle vs paclitaxel groups. All sections were counterstained with DAPI (blue). Data represented as mean and SEM. Two-way ANOVA was performed with Sidak’s post-hoc for multiple comparisons; n=3 for all groups. Magnification 20X, scale bar 50µm. PTX – paclitaxel.

Previous studies have shown no association between sickness behavior or change in the body weight of the animal with 16mg/Kg dose of paclitaxel. Moreover, other researchers have used even higher doses of paclitaxel to induce peripheral neuropathy (57, 58). We assessed number of DRG neurons positive for activating transcription factor 3 (ATF3). The number of ATF3^+^ neurons were significantly higher in the DRG of paclitaxel-treated animals indicating damage; however, there were no sex-based or genotype differences observed (**Figure 6** and **Table 5**). The increase is similar to previously published literature, albeit at a later time point (59, 60), thus we conclude that this higher dose does not comparatively alter DRG neurons at day 10 after our regimen.

**Table 5:** Statistical values for IHC data analysis.

Dataset	Main effect		Interaction		Multiple comparisons		
	F (DFn,DFd)	*p*-value	F (DFn,DFd)	*p*-value	Effect	Groups	*p*-value
Male mice ATF3^+^ cells	Treatment: F (1, 8) = 10.67	**0.0114**	F (1, 8) = 2.042	0.1909	Treatment	Vehicle	0.6539
						Paclitaxel	0.4838
	Genotype: F (1, 8) = 0.04	0.8434			Genotype	WT	0.4073
						eIF4E^S209A^	**0.0210**
Female mice ATF3^+^ cells	Treatment: F (1, 8) = 8.067	**0.0218**	F (1, 8) = 0.600	0.4609	Treatment	Vehicle	0.9803
						Paclitaxel	0.4179
	Genotype: F (1, 8) = 1.067	0.3319			Genotype	WT	0.0666
						eIF4E^S209A^	0.3313
Male mice Iba1	Treatment: F (1, 8) = 10.06	**0.0132**	F (1, 8) = 6.556	**0.0336**	Treatment	Vehicle	>0.9999
						Paclitaxel	**0.0140**
	Genotype: F (1, 8) = 13.24	**0.0066**			Genotype	WT	**0.0220**
						eIF4E^S209A^	>0.9999
Female mice Iba1	Treatment: F (1, 6) = 7.540	**0.0335**	F (1, 6) = 18.22	**0.0053**	Treatment	Vehicle	0.9194
						Paclitaxel	**0.0029**
	Genotype: F (1, 6) = 40.14	**0.00071**			Genotype	WT	**0.0153**
						eIF4E^S209A^	>0.9999
Male mice GFAP	Treatment: F (1, 8) = 12.90	**0.0071**	F (1, 8) = 7.104	**0.0286**	Treatment	Vehicle	**0.0133**
						Paclitaxel	>0.9999
	Genotype: F (1, 8) = 1.075	0.3301			Genotype	WT	>0.9999
						eIF4E^S209A^	0.1845
Female mice GFAP	Treatment: F (1, 6) = 40.81	**0.0007**	F (1, 6) = 0.1627	0.7007	Treatment	Vehicle	**0.0180**
						Paclitaxel	**0.0329**
	Genotype: F (1, 6) = 2.078	0.1995			Genotype	WT	>0.9999
						eIF4E^S209A^	>0.9999

Two-way ANOVA was performed followed by Sidak’s post-hoc for ATF3^+^ cell number and Bonferroni’s post-hoc for Iba1 and GFAP for multiple comparisons between genotypes and treatments for each sex. p ≤ 0.05 was considered significant (bolded).

### Spinal Glia Reactivity Is Altered in a Sex-Dependent Manner With Paclitaxel Treatment

**Figure 7:**
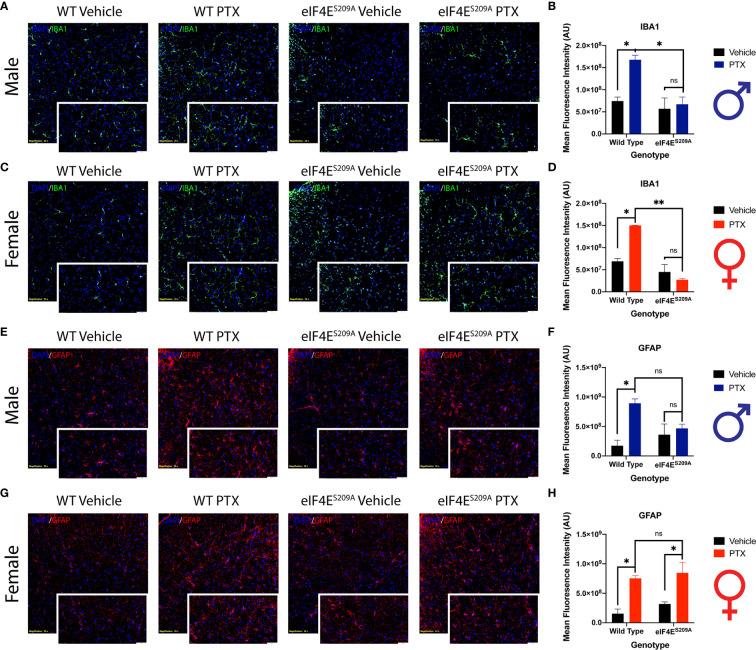
Spinal glia reactivity is altered in a sex-dependent manner with paclitaxel treatment. **(A)** Immunohistochemistry for male microglia on lumbar spinal cord. Blue represents DAPI for cell nuclei, green represents Iba1 for microglia. Magnification 20X, scale bar 50µm. **(B)** Quantification of Iba1 fluorescence intensity from male mice, *p 0.022 for WT vehicle vs WT paclitaxel groups, *p 0.014 for WT paclitaxel vs eIF4E^S209A^ paclitaxel groups. **(C)** Immunohistochemistry for female microglia on lumbar spinal cord. Blue represents DAPI for cell nuclei, green represents Iba1 for microglia. Magnification 20X, scale bar 50µm. **(D)** Quantification of Iba1 fluorescence intensity from female mice, *p 0.0153, **p 0.0029. **(E)** Immunohistochemistry for male astrocytes on lumbar spinal cord. Blue represents DAPI for cell nuclei, red represents GFAP for astrocytes. Magnification 20X, scale bar 50µm. **(F)** Quantification of GFAP fluorescence intensity from male mice, *p 0.0133. **(G)** Immunohistochemistry for female astrocytes on lumbar spinal cord. Blue represents DAPI for cell nuclei, red represents GFAP for astrocytes. Magnification 20X, scale bar 50µm. **(H)** Quantification of GFAP fluorescence intensity from female mice, *p 0.018 for WT vehicle vs WT paclitaxel groups, *p 0.0329 for eIF4E^S209A^ vehicle vs eIF4E^S209A^ paclitaxel groups. Data are presented as mean ± standard error of the mean, males: n = 3 mice per group; females n = 3 mice per group. Two-way ANOVA was performed with Bonferroni’s *post-hoc* for multiple comparisons. ns, not significant.

Previous data has shown activation of microglia and astrocytes at different phases of CIPN (61–63). Here, we investigated whether the eIF4E complex plays a role in the activation of microglia and astrocytes in the spinal cord. We found a significant increase in immunoreactivity of microglia and astrocytes in both male and female WT mice (**Figure 7**). Iba1 fluorescence was significantly higher in paclitaxel-treated WT mice of both sexes compared to vehicle (**Figures 7A–C** and **Table 5**), indicating that paclitaxel induced microglial activation. This increase was lacking for eIF4E^S209A^ animals for whom the Iba1 immunoreactivity stayed at WT vehicle levels. In eIF4E^S209A^ females, there was a decrease in Iba1 fluorescence with paclitaxel treatment compared to vehicle (**Figure 7C**). For astrocytes, females lacking cap-dependent translation had increased GFAP fluorescence after paclitaxel compared to vehicle (**Figures 7D, F, G, and H**, and **Table 5**), which was different from the males where the GFAP fluorescence was at the same level as seen for the WT vehicle group (**Figures 7D, E**). This data shows that paclitaxel may mediate activation of astrocytes via an eIF4E-independent pathway in females but modulates microglia activation via eIF4E in both sexes and astrocyte activation in males.

## Discussion

The current study begins to uncover the importance of eIF4E phosphorylation in the development of CIPN and associated neuroimmune consequences in male and female mice. In our study, we found an eIF4E-dependent increase of mechanical and thermal hypersensitivity in both sexes, upregulated T-helper and reduced cytotoxic T-lymphocytes in draining lymph nodes in female but not male mice, whereas activated macrophages in DRG were significantly increased in eIF4E but not WT mice of both sexes. In addition to this, DRG neuronal mitochondrial function was significantly higher in WT mice but not in eIF4E mice of both sexes. Microglia were significantly activated in an eIF4E-dependent manner with paclitaxel treatment in WT mice of both sexes; however, astrocytes were activated in an eIF4E-dependent manner with paclitaxel treatment only in male mice. Female eIF4E mice showed significant astrocyte activation.

Our study was designed and powered with the intent to appropriately assess sex differences in neuroimmune interactions during CIPN development—a question left unanswered in previous literature. We found an eIF4E-dependent increase in spontaneous pain as well as mechanical and thermal hypersensitivity after paclitaxel. This suggests that the pain-like behavior changes due to paclitaxel are mediated via the central cap-dependent translation pathway that is common across diverse cell types, with more pronounced effects in female mice.

The cell bodies of primary afferent neurons reside in the dorsal root ganglia. The persistent signals from nerve terminals activate the machinery and signaling cascades in the nucleus and cytoplasm of the neuronal soma (64–66). Hyper-excited nociceptor neurons recruit immune cells by secreting chemokines, neurotransmitters, and/or other neuropeptides (16, 67). It has been shown that paclitaxel treatment recruits macrophages into the DRG (20). Our findings corroborate this finding at an earlier time point and add to this knowledge by characterizing the macrophage population as CD45^+^CD11b^+^CD40^+^MHC-II^+^ i.e. activated APCs. It has been shown that such infiltration may be beneficial to recruit Th2 anti-inflammatory cells at later time points (62, 68). We have presented evidence that paclitaxel injection leads to a significant increase in the activated APC population in eIF4E males and females, but not in WT mice. Thus, a lack of eIF4E phosphorylation or cap-dependent translation exacerbates the immune cell response and DRG infiltration during CIPN development.

A prior study performed only in male mice showed an increase in CD4^+^ T-cells at paclitaxel post-injection day 7 (14). We found a similar increase of CD4^+^ T-cells in female WT mice with paclitaxel treatment. Additionally, a novel finding was that CD8^+^ T-cells were decreased in female but not in male WT mice during early phase of CIPN development. This indicates sex differences in T-cell activation in the lymph nodes, which would affect the subsets of T-cells that would infiltrate other tissues such as DRG (69). A previously published study showed increased numbers of CD3^+^, helper T-cells, and cytotoxic T-lymphocytes after paclitaxel on day 7 in mice DRG (70). Our dataset adds niche T-cell population characterization to this previously published literature. T-cell subset activation in the lymph nodes is highly dependent upon the antigen-presenting cells naïve T-cells encounter (71). Once activated, these subsets follow their divergent fates of redistribution to tissues in need of immune response. Thus, characterizing different subsets of T-cells in the lymph nodes allows us to determine what populations we can expect in interfaces where neuroimmune communication can occur, such as the meninges or DRG. T-cells are also important in the transition from acute to chronic pain (15). By showing alterations in specialized T-cell subsets, our study raises important questions about the implications for establishment of CIPN and its resolution. Animals lacking eIF4E had higher proportion of memory T-cells in both sexes. There were no changes in the effector T-cell population for the lymph nodes. These alterations in the activation and proportion of T-cell subsets and APCs suggest that although affective pain behavior is similar in male and female mice after development of CIPN, the mechanism driving the pain phenotypes in both sexes involves varied contribution from innate and adaptive immune cells.

Basal respiration and non-mitochondrial respiration were higher in male DRG neurons treated with paclitaxel compared to vehicle, which is contrary to a previous report for rats (38). We have further shown that the lack of eIF4E does not change this trend, even though oxygen consumption is lower, suggesting that paclitaxel may induce mitochondrial function independent of cap-dependent translation regulation. In females, while the effects on basal respiration follow the same trend as the males, paclitaxel seems to mediate an increase in non-mitochondrial respiration independent of eIF4E. Female DRG neurons also had significantly higher ATP turnover and maximal respiration with paclitaxel treatment in WT. Considering that eIF4E female mice show higher mechanical hypersensitivity with paclitaxel and that their DRG neuron ATP turnover and maximal respiration is higher, this may be indicative of the severity of the pain phenotype. Whereas for males, the increased basal respiration may reflect the pain phenotype in general. To our knowledge, this is the first study to show sex differences in mitochondrial bioenergetics in the premise of cap-dependent translation for paclitaxel-induced peripheral neuropathy. These differences seen early during CIPN development, may be important for identifying strategies to prevent its establishment in both sexes. Thus, it is necessary to understand the particular mitochondrial pathways downstream of eIF4E to identify potential targets to prevent CIPN. Alternatively, therapies that address restoration of normal mitochondrial function and morphology are also promising for either sex.

Previously published data has shown that paclitaxel leads to spinal activation of astrocytes but not microglia at different phases of paclitaxel-induced peripheral neuropathy in mouse and rat models (72, 73). In contrast, we found that both microglia and astrocytes are significantly activated after paclitaxel injection in male mice, which is eIF4E dependent. However, the astrocyte reactivity increase in female mice was independent of eIF4E, suggesting that paclitaxel may have alternate mechanisms of astrocyte activation in females. Activated glia are known to secrete a number of pro-inflammatory mediators that act directly on CNS neurons, which can sensitize them if subjected to constant stimulation over extended periods (64). This has implications for the establishment of pain phenotypes post paclitaxel treatment. It has also been shown that intraperitoneal paclitaxel can activate astrocytes and mediate mechanical allodynia in the 1^st^ hour, possibly by miniscule amounts crossing the blood-brain barrier (62). Thus, the glia activation seen 10 days post our paclitaxel regimen may be important for onset as well as establishment of CIPN through cap-dependent translation in males but via other pathways in females.

Regulation of cap-dependent translation happens via multiple different pathways. This is partly related to the expression of cell-specific surface receptors such as TrkA/B, gp130, mGLuR1/5, and insulin receptors and this influences which pathway is activated in response to which stimuli (74–76). In behavioral experiments, we assessed the role of eIf4E for regulation of brain behaviors i.e the interaction of all cells in the body after insult, but in the molecular experiments, we target specific populations of cells to understand how they individually change as a result of eIF4E manipulation and paclitaxel treatment. Our study provides novel insight into how cap-dependent translation dysregulation of immune or neuronal cells may be important for sex-divergent mechanisms of early CIPN. But comprehensive future studies would still be required to further deduce the cell-specific responses after paclitaxel treatment in the absence of cap-dependent translation.

Overall, our data suggest that although pain-like behavior during the early development of the CIPN are similar, the mechanisms and cell types involved in engendering these behaviors are different in males and females. The differences in immune cell populations and activation of glia indicate that separate upstream pathways may regulate cap-dependent translation in both sexes and thus lead to changed downstream outcomes. The central player i.e. the eIF4E complex can thus be a valuable target for preventing establishment of CIPN and limiting associated pain.

## Data Availability Statement

The raw data supporting the conclusions of this article will be made available by the authors, without undue reservation.

## Ethics Statement

The animal study was reviewed and approved by University of Texas at Dallas Institute for Animal Care and Use Committee (IACUC).

## Author Contributions

Methodology, NA. Data analysis and curation, NA, PM, TS-P, and HJ. Writing manuscript and drawing figures, NA, PM, TS-P, and HJ. MB conceptualized the study and participated and supervised in all aspects of the study and manuscript preparation. All authors contributed to the article and approved the submitted version.

## Funding

This research was funded by the NIH/NINDS, grant number K22NS096030 (MB), the University of Texas System STARS program research support grant (MB), the American Pain Society Future Leaders Grant (MB), and the Rita Allen Foundation Grant (MB).

## Conflict of Interest

The authors declare that the research was conducted in the absence of any commercial or financial relationships that could be construed as a potential conflict of interest.
