# Supplementary material for: Neuroimmune Consequences of eIF4E Phosphorylation on Chemotherapy-Induced Peripheral Neuropathy
Source: Front Immunol. 2021 Apr 12;12:642420. doi: 10.3389/fimmu.2021.642420 (PMC8071873; doi:10.3389/fimmu.2021.642420)
Supplement: Supplementary file 1 [file DataSheet_1.docx]

Table 1*.* Antibodies used for IHC and flow cytometry.

| **Antibody** | **Company** | **Catalog number** | **Working dilution** |
| --- | --- | --- | --- |
| *Antibodies used for IHC* | | | |
| Anti-Iba1 | WAKO | 019-19741 | 1:1000 |
| Anti-GFAP | DAKO | Z-0331 | 1:1000 |
| Anti-NeuN | EMD Millipore | MAB377 | 1:1000 |
| Anti-ATF3 | Abcam | ab207434 | 1:1000 |
| Goat anti-mouse Alexa Fluor 488 | Invitrogen | A21121 | 1:500 |
| Goat anti-rabbit Alexa Fluor 647 | Invitrogen | A21245 | 1:500 |
| *Antibodies used for flow cytometry* | | | |
| Anti-CD16/32 | eBioscience | 16016185 | 1:2000 |
| Anti-CD3 Alexa fluor 700 conjugate | eBioscience | 56003280 | 1:200 |
| Anti-CD4 Fluorescein isothiocyanate conjugate | eBioscience | 11004185 | 1:200 |
| Anti-CD8 Phycoerythrin conjugate | eBioscience | 12-0081-83 | 1:200 |
| Anti-CD25 eFluor 450 conjugate | eBioscience | 48025182 | 1:200 |
| Anti-CD44 eFluor780 conjugate | eBioscience | 47044182 | 1:200 |
| Anti-CCR7 Allophycocyanin conjugate | eBioscience | 17197942 | 1:200 |
| Anti-CD11b Allophycocyanin-Cy7 conjugate | Life Technologies | A15390 | 1:200 |
| Anti-CD45 Brilliant violet 421 conjugate | Biolegend | 103133 | 1:200 |
| Anti-MHCII Alexa fluor 488 conjugate | eBioscience | 11532282 | 1:2000 |
| Anti-CD40 Phycoerythrin conjugate | eBioscience | 120401-82 | 1:200 |

**Table 2. Statistical values for behavior data analysis.** Two-way ANOVA was performed followed by Tukey’s *post-hoc* for multiple comparisons between genotypes and treatments for each sex. *p* ≤ 0.05 was considered significant (bolded).

| **Dataset** | **Main effect** | | **Interactions** | | **Multiple comparisons** | | |
| --- | --- | --- | --- | --- | --- | --- | --- |
|  | **F(DFn,DFd)** | ***p*-value** | **F(DFn,DFd)** | ***p*-value** | **Effect** | **Groups** | ***p*-value** |
| Mechanical hypersensitivity male mice | Treatment: F (1, 25) = 12.26 | **0.0018** | F (1, 25) = 9.797 | **0.0044** | Treatment | Vehicle | 0.9938 |
|  |  |  |  |  |  | Paclitaxel | **0.0004** |
|  | Genotype: F (1, 25) = 26.48 | **<0.0001** |  |  | Genotype | WT | **<0.0001** |
|  |  |  |  |  |  | eIF4E^S209A^ | 0.5380 |
| Mechanical hypersensitivity female mice | Treatment: F (1, 19) = 251.1 | **<0.0001** | F (1, 19) = 24.49 | **<0.0001** | Treatment | Vehicle | 0.8600 |
|  |  |  |  |  |  | Paclitaxel | **<0.0001** |
|  | Genotype: F (1, 19) = 36.87 | **<0.0001** |  |  | Genotype | WT | **<0.0001** |
|  |  |  |  |  |  | eIF4E^S209A^ | **<0.0001** |
| Grimace male mice | Treatment: F (1, 15) = 4.236 | **0.0574** | F (1, 15) = 5.111 | **0.0391** | Treatment | Vehicle | 0.8146 |
|  |  |  |  |  |  | Paclitaxel | 0.1282 |
|  | Genotype: F (1, 15) = 0.9575 | 0.3433 |  |  | Genotype | WT | **0.0302** |
|  |  |  |  |  |  | eIF4E^S209A^ | 0.9990 |
| Grimace female mice | Treatment: F (1, 19) = 34.43 | **<0.0001** | F (1, 19) = 4.609 | **0.0449** | Treatment | Vehicle | >0.9999 |
|  |  |  |  |  |  | Paclitaxel | **0.0293** |
|  | Genotype: F (1, 19) = 4.609 | **0.0449** |  |  | Genotype | WT | **0.0005** |
|  |  |  |  |  |  | eIF4E^S209A^ | **0.0249** |
| Grip strength male mice | Treatment: F (1, 18) = 0.6734 | 0.4226 | F (1, 18) = 0.2471 | 0.6251 | N/A | | |
|  | Genotype: F (1, 18) = 0.7916 | 0.3854 |  |  |  |  |  |
| Grip strength female mice | Treatment: F (1, 16) = 0.8995 | 0.3570 | F (1, 16) = 0.8995 | 0.3570 | N/A | | |
|  | Genotype: F (1, 16) = 2.349 | 0.1449 |  |  |  |  |  |
| Thermal hypersensitivity male mice | Treatment: F (1, 24) = 6.294 | **0.0193** | F (1, 24) = 6.743 | **0.0158** | Treatment | Vehicle | 0.7602 |
|  |  |  |  |  |  | Paclitaxel | **0.0579** |
|  | Genotype: F (1, 24) = 1.455 | 0.2395 |  |  | Genotype | WT | **0.0072** |
|  |  |  |  |  |  | eIF4E^S209A^ | >0.9999 |
| Thermal hypersensitivity female mice | Treatment: F (1, 19) = 2.332 | 0.1432 | F (1, 19) = 1.090 | 0.3097 | Treatment | Vehicle | 0.5527 |
|  |  |  |  |  |  | Paclitaxel | **0.0453** |
|  | Genotype: F (1, 19) = 8.729 | **0.0081** |  |  | Genotype | WT | 0.4059 |
|  |  |  |  |  |  | eIF4E^S209A^ | 0.9762 |

**Table 3. Statistical values for flow cytometry data analysis.** Two-way ANOVA was performed followed by Tukey’s *post-hoc* for multiple comparisons between genotypes and treatments for each sex. *p* ≤ 0.05 was considered significant (bolded).

| **Dataset** | **Main effect** | | **Interactions** | | **Multiple comparisons** | | |
| --- | --- | --- | --- | --- | --- | --- | --- |
|  | **F(DFn,DFd)** | ***p*-value** | **F(DFn,DFd)** | ***p*-value** | **Effect** | **Groups** | ***p*-value** |
| *Flow cytometry data analysis for T-cells from popliteal and inguinal lymph nodes* | | | | | | | |
| CD4^+^ males | Treatment: F (1, 10) = 3.139 | 0.1069 | F (1, 10) = 0.3061 | 0.5922 | N/A | | |
|  | Genotype: F (1, 10) = 0.6463 | 0.4401 |  |  |  |  |  |
| CD4^+^CCR7^+^ males | Treatment: F (1, 10) = 0.1196 | 0.7367 | F (1, 10) = 0.1991 | 0.6650 | Treatment | Vehicle | 0.0814 |
|  |  |  |  |  |  | Paclitaxel | 0.2116 |
|  | Genotype: F (1, 10) = 11.86 | **0.0063** |  |  | Genotype | WT | 0.9514 |
|  |  |  |  |  |  | eIF4E^S209A^ | 0.9998 |
| CD4^+^CD44^±^CD25^±^ males | Cell populations: F (2, 30) = 809.1 | **<0.0001** | F (6, 30) = 0.2916 | 0.9362 | No genotype or treatment difference within CD4^+^CD44^+^CD25^-^, CD4^+^CD44^-^CD25^+^, or CD4^+^CD44^+^CD25^+^ populations. | | |
|  | Genotype-treatment: F (3, 30) = 0.6686 | 0.5780 |  |  |  |  |  |
| CD4^+^ females | Treatment: F (1, 10) = 17.44 | **0.0019** | F (1, 10) = 3.920 | 0.0759 | Treatment | Vehicle | 0.3920 |
|  |  |  |  |  |  | Paclitaxel | 0.6742 |
|  | Genotype: F (1, 10) = 0.1340 | 0.7219 |  |  | Genotype | WT | **0.0101** |
|  |  |  |  |  |  | eIF4E^S209A^ | 0.3831 |
| CD4^+^CCR7^+^ females | Treatment: F (1, 10) = 0.0027 | 0.9591 | F (1, 10) = 0.0341 | 0.8571 | Treatment | Vehicle | 0.3583 |
|  |  |  |  |  |  | Paclitaxel | 0.2532 |
|  | Genotype: F (1, 10) = 6.933 | **0.0250** |  |  | Genotype | WT | 0.9985 |
|  |  |  |  |  |  | eIF4E^S209A^ | 0.9996 |
| CD4^+^CD44^±^CD25^±^ females | Cell populations: F (2, 30) = 1893 | **<0.0001** | F (6, 30) = 2.406 | 0.0512 | CD4^+^CD44^+^CD25^-^ Genotype ns | | |
|  | Genotype-treatment: F (3, 30) = 2.452 | 0.0827 |  |  | CD4^+^CD44^+^CD25^-^ Treatment | Vehicle | **0.0011** |
|  |  |  |  |  |  | Paclitaxel | 0.7767 |
| CD8^+^ males | Treatment: F (1, 10) = 3.180 | 0.1049 | F (1, 10) = 0.5157 | 0.4891 | N/A | | |
|  | Genotype: F (1, 10) = 0.6004 | 0.4564 |  |  |  |  |  |
| CD8^+^CCR7^+^ males | Treatment: F (1, 10) = 0.0917 | 0.7681 | F (1, 10) = 0.1555 | 0.7016 | Treatment | Vehicle | **0.0433** |
|  |  |  |  |  |  | Paclitaxel | 0.1044 |
|  | Genotype: F (1, 10) = 16.48 | **0.0023** |  |  | Genotype | WT | >0.9999 |
|  |  |  |  |  |  | eIF4E^S209A^ | 0.9491 |
| CD8^+^CD44^±^CD25^±^ males | Cell populations: F (2, 30) = 772.5 | **<0.0001** | F (6, 30) = 1.591 | 0.1842 | CD8^+^CD44^+^CD25^-^ Genotype ns | | |
|  | Genotype-treatment: F (3, 30) = 1.096 | 0.3660 |  |  | CD8^+^CD44^+^CD25^-^Treatment | Vehicle | 0.1579 |
|  |  |  |  |  |  | Paclitaxel | **0.0396** |
| CD8^+^ females | Treatment: F (1, 10) = 5.496 | **0.0410** | F (1, 10) = 3.684 | 0.0839 | Treatment | Vehicle | 0.6174 |
|  |  |  |  |  |  | Paclitaxel | 0.4863 |
|  | Genotype: F (1, 10) = 0.0273 | 0.8720 |  |  | Genotype | WT | 0.0730 |
|  |  |  |  |  |  | eIF4E^S209A^ | 0.9875 |
| CD8^+^CCR7^±^ females | Treatment: F (1, 10) = 0.1574 | 0.6999 | F (1, 10) = 0.0371 | 0.8510 | Treatment | Vehicle | 0.1422 |
|  |  |  |  |  |  | Paclitaxel | 0.2125 |
|  | Genotype: F (1, 10) = 10.15 | **0.0097** |  |  | Genotype | WT | 0.9991 |
|  |  |  |  |  |  | eIF4E^S209A^ | 0.9681 |
| CD8^+^CD44^±^CD25^±^ females | Cell populations: F (2, 30) = 788.2 | **<0.0001** | F (6, 30) = 0.7166 | 0.6392 | No genotype or treatment difference within CD8^+^CD44^+^CD25^-^, CD8^+^CD44^-^CD25^+^, or CD8^+^CD44^+^CD25^+^ populations. | | |
|  | Genotype-treatment: F (3, 30) = 0.5383 | 0.6597 |  |  |  |  |  |
| *Flow cytometry data analysis for myeloid cells from DRGDRG* | | | | | | | |
| Males CD11b^+^CD45^+^CD40^±^MHCII^±^ | Cell populations: F (2, 24) = 84.59 | **<0.0001** | F (6, 24) = 1.803 | 0.1410 | CD40^+^MHCII^+^ Treatment ns | | |
|  | Genotype-treatment: F (3, 24) = 1.139 | 0.3535 |  |  | CD40^+^MHCII^+^ Genotype | WT | 0.4651 |
|  |  |  |  |  |  | eIF4E^S209A^ | **0.0275** |
| Females CD11b^+^CD45^+^CD40^±^MHCII^±^ | Cell populations: F (2, 24) = 141.3 | **<0.0001** | F (6, 24) = 4.720 | **0.0026** | CD40^+^MHCII^+^ Treatment | Vehicle | **0.0140** |
|  |  |  |  |  |  | Paclitaxel | **0.0055** |
|  | Genotype-treatment: F (3, 24) = 5.042 | **0.0075** |  |  | CD40^+^MHCII^+^ Genotype | WT | 0.5182 |
|  |  |  |  |  |  | eIF4E^S209A^ | **<0.0001** |

**Table 4. Statistical values for DRG neuron mitochondrial respiration.** Two-way ANOVA was performed on AUC values for OCR and ECAR followed by Tukey’s *post-hoc* for multiple comparisons between genotypes and treatments for each sex. For breakdown data of mitochondrial respiration, ordinary one-way ANOVAs were used to analyze mean values of four groups (WT vehicle, WT paclitaxel, eIF4E^S209A^ vehicle, and eIF4ES^209A^ paclitaxel), followed by Sidak’s *post-hoc* for multiple comparisons between genotypes and treatments for each sex. *p* ≤ 0.05 was considered significant (bolded). OCR – oxygen consumption rate, ECAR – extracellular acidification rate.

| **Dataset** | **Main effect** | | **Interaction** | | **Multiple comparisons** | | | |
| --- | --- | --- | --- | --- | --- | --- | --- | --- |
|  | **F(DFn,DFd)** | **p-value** | **F(DFn,DFd)** | **p-value** | **Effect** | | **Groups** | **p-value** |
| Males OCR AUC | Treatment: F (1, 11) = 11.85 | **0.0055** | F (1, 11) = 5.817 | **0.0345** | Treatment | | Vehicle | 0.5347 |
|  |  |  |  |  |  |  | Paclitaxel | 0.2303 |
|  | Genotype: F (1, 11) = 0.1714 | 0.6868 |  |  | Genotype | | WT | **0.0136** |
|  |  |  |  |  |  |  | eIF4E^S209A^ | 0.8475 |
| Females OCR AUC | Treatment: F (1, 17) = 6.074 | **0.0247** | F (1, 17) = 5.158 | **0.0364** | Treatment | | Vehicle | 0.7519 |
|  |  |  |  |  |  |  | Paclitaxel | 0.1426 |
|  | Genotype: F (1, 17) = 0.6271 | 0.4393 |  |  | Genotype | | WT | **0.0455** |
|  |  |  |  |  |  |  | eIF4E^S209A^ | 0.9982 |
| Males ECAR AUC | Treatment: F (1, 11) = 13.04 | **0.0041** | F (1, 11) = 16.26 | **0.0020** | Treatment | | Vehicle | 0.1313 |
|  |  |  |  |  |  |  | Paclitaxel | **0.0303** |
|  | Genotype: F (1, 11) = 0.2927 | 0.5993 |  |  | Genotype | | WT | **0.0021** |
|  |  |  |  |  |  |  | eIF4E^S209A^ | 0.9866 |
| Females ECAR AUC | Treatment: F (1, 17) = 7.989 | **0.0116** | F (1, 17) = 2.068 | 0.1686 | Treatment | | Vehicle | 0.6269 |
|  |  |  |  |  |  |  | Paclitaxel | 0.8523 |
|  | Genotype: F (1, 17) = 0.1287 | 0.7242 |  |  | Genotype | | WT | 0.0792 |
|  |  |  |  |  |  |  | eIF4E^S209A^ | 0.6287 |
| **Breakdown datasets for OCR** | **Interaction** | **p-value** | **Multiple comparisons - groups compared** | | | | | **p-value** |
| Males basal respiration | F (3, 8) = 233.2 | **<0.0001** | WT vehicle | | WT paclitaxel | | | **<0.0001** |
|  |  |  | eIF4E^S209A^ vehicle | | eIF4E^S209A^ paclitaxel | | | **0.0134** |
|  |  |  | WT vehicle | | eIF4E^S209A^ vehicle | | | **0.0018** |
|  |  |  | WT paclitaxel | | eIF4E^S209A^ paclitaxel | | | **<0.0001** |
| Females basal respiration | F (3, 8) = 838.7 | **<0.0001** | WT vehicle | | WT paclitaxel | | | **<0.0001** |
|  |  |  | eIF4E^S209A^ vehicle | | eIF4E^S209A^ paclitaxel | | | **<0.0001** |
|  |  |  | WT vehicle | | eIF4E^S209A^ vehicle | | | 0.8413 |
|  |  |  | WT paclitaxel | | eIF4E^S209A^ paclitaxel | | | **<0.0001** |
| Males ATP turnover | F (3, 12) = 2.384 | 0.1203 | WT vehicle | | WT paclitaxel | | | 0.0905 |
|  |  |  | eIF4E^S209A^ vehicle | | eIF4E^S209A^ paclitaxel | | | 0.9832 |
|  |  |  | WT vehicle | | eIF4E^S209A^ vehicle | | | 0.9188 |
|  |  |  | WT paclitaxel | | eIF4E^S209A^ paclitaxel | | | 0.5864 |
| Females ATP turnover | F (3, 12) = 8.828 | **0.0023** | WT vehicle | | WT paclitaxel | | | 0.0021 |
|  |  |  | eIF4E^S209A^ vehicle | | eIF4E^S209A^ paclitaxel | | | 0.6736 |
|  |  |  | WT vehicle | | eIF4E^S209A^ vehicle | | | 0.9766 |
|  |  |  | WT paclitaxel | | eIF4E^S209A^ paclitaxel | | | 0.0481 |
| Males maximal respiration | F (3, 28) = 2.233 | 0.1064 | WT vehicle | | WT paclitaxel | | | 0.0661 |
|  |  |  | eIF4E^S209A^ vehicle | | eIF4E^S209A^ paclitaxel | | | 0.9857 |
|  |  |  | WT vehicle | | eIF4E^S209A^ vehicle | | | 0.8539 |
|  |  |  | WT paclitaxel | | eIF4E^S209A^ paclitaxel | | | 0.6683 |
| Females maximal respiration | F (3, 28) = 5.986 | **0.0028** | WT vehicle | | WT paclitaxel | | | **0.0011** |
|  |  |  | eIF4E^S209A^ vehicle | | eIF4E^S209A^ paclitaxel | | | >0.9999 |
|  |  |  | WT vehicle | | | eIF4E^S209A^ vehicle | | 0.4836 |
|  |  |  | WT paclitaxel | | | eIF4E^S209A^ paclitaxel | | **0.0417** |
| Males non-mitochondrial respiration (reserve capacity) | F (3, 8) = 42.18 | **<0.0001** | WT vehicle | | | WT paclitaxel | | **<0.0001** |
|  |  |  | eIF4E^S209A^ vehicle | | | eIF4E^S209A^ paclitaxel | | 0.9978 |
|  |  |  | WT vehicle | | | eIF4E^S209A^ vehicle | | **0.0063** |
|  |  |  | WT paclitaxel | | | eIF4E^S209A^ paclitaxel | | **0.0010** |
| Females non-mitochondrial respiration (reserve capacity) | F (3, 8) = 46.31 | **<0.0001** | WT vehicle | | | WT paclitaxel | | **<0.0001** |
|  |  |  | eIF4E^S209A^ vehicle | | | eIF4E^S209A^ paclitaxel | | **0.0074** |
|  |  |  | WT vehicle | | | eIF4E^S209A^ vehicle | | **0.0037** |
|  |  |  | WT paclitaxel | | | eIF4E^S209A^ paclitaxel | | 0.9212 |

**Table 5. Statistical values for IHC data analysis.** Two-way ANOVA was performed followed by Sidak’s *post-hoc* for ATF3^+^ cell number and Bonferroni’s *post-hoc* for Iba1 and GFAP for multiple comparisons between genotypes and treatments for each sex. *p* ≤ 0.05 was considered significant (bolded).

| **Dataset** | **Main effect** | | **Interaction** | | **Multiple comparisons** | | |
| --- | --- | --- | --- | --- | --- | --- | --- |
|  | **F(DFn,DFd)** | ***p*-value** | **F(DFn,DFd)** | ***p*-value** | **Effect** | **Groups** | ***p*-value** |
| Male mice ATF3^+^ cells | Treatment: F (1, 8) = 10.67 | **0.0114** | F (1, 8) = 2.042 | 0.1909 | Treatment | Vehicle | 0.6539 |
|  |  |  |  |  |  | Paclitaxel | 0.4838 |
|  | Genotype: F (1, 8) = 0.04 | 0.8434 |  |  | Genotype | WT | 0.4073 |
|  |  |  |  |  |  | eIF4E^S209A^ | **0.0210** |
| Female mice ATF3^+^ cells | Treatment: F (1, 8) = 8.067 | **0.0218** | F (1, 8) = 0.600 | 0.4609 | Treatment | Vehicle | 0.9803 |
|  |  |  |  |  |  | Paclitaxel | 0.4179 |
|  | Genotype: F (1, 8) = 1.067 | 0.3319 |  |  | Genotype | WT | 0.0666 |
|  |  |  |  |  |  | eIF4E^S209A^ | 0.3313 |
| Male mice Iba1 | Treatment: F (1, 8) = 10.06 | **0.0132** | F (1, 8) = 6.556 | **0.0336** | Treatment | Vehicle | >0.9999 |
|  |  |  |  |  |  | Paclitaxel | **0.0140** |
|  | Genotype: F (1, 8) = 13.24 | **0.0066** |  |  | Genotype | WT | **0.0220** |
|  |  |  |  |  |  | eIF4E^S209A^ | >0.9999 |
| Female mice Iba1 | Treatment: F (1, 6) = 7.540 | **0.0335** | F (1, 6) = 18.22 | **0.0053** | Treatment | Vehicle | 0.9194 |
|  |  |  |  |  |  | Paclitaxel | **0.0029** |
|  | Genotype: F (1, 6) = 40.14 | **0.00071** |  |  | Genotype | WT | **0.0153** |
|  |  |  |  |  |  | eIF4E^S209A^ | >0.9999 |
| Male mice GFAP | Treatment: F (1, 8) = 12.90 | **0.0071** | F (1, 8) = 7.104 | **0.0286** | Treatment | Vehicle | **0.0133** |
|  |  |  |  |  |  | Paclitaxel | >0.9999 |
|  | Genotype: F (1, 8) = 1.075 | 0.3301 |  |  | Genotype | WT | >0.9999 |
|  |  |  |  |  |  | eIF4E^S209A^ | 0.1845 |
| Female mice GFAP | Treatment: F (1, 6) = 40.81 | **0.0007** | F (1, 6) = 0.1627 | 0.7007 | Treatment | Vehicle | **0.0180** |
|  |  |  |  |  |  | Paclitaxel | **0.0329** |
|  | Genotype: F (1, 6) = 2.078 | 0.1995 |  |  | Genotype | WT | >0.9999 |
|  |  |  |  |  |  | eIF4E^S209A^ | >0.9999 |
