# Supplementary figures and images for: Neuroimmune Consequences of eIF4E Phosphorylation on Chemotherapy-Induced Peripheral Neuropathy
Source: Front Immunol. 2021 Apr 12;12:642420. doi: 10.3389/fimmu.2021.642420 (PMC8071873; doi:10.3389/fimmu.2021.642420)

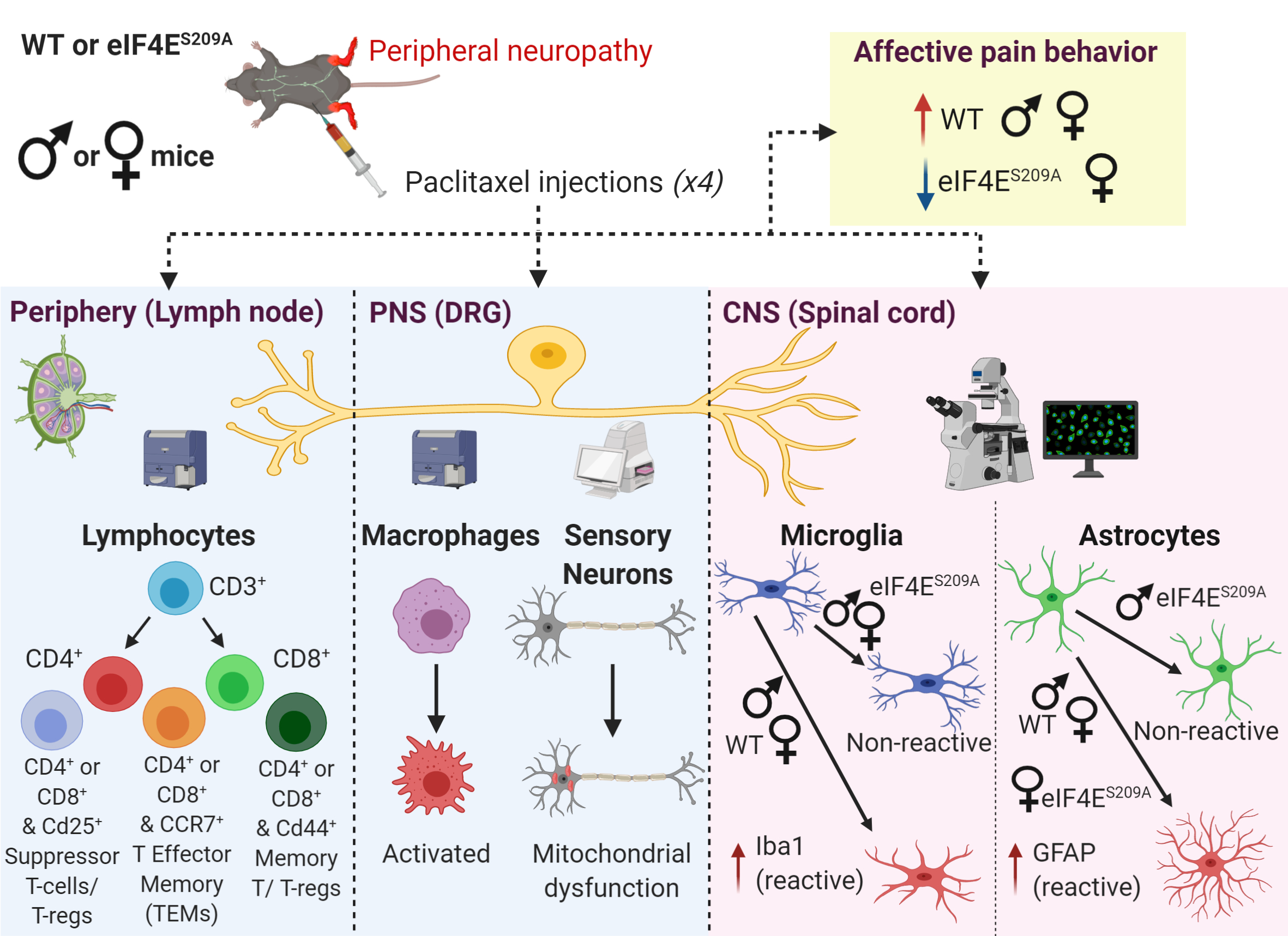

Supplement: Supplementary file 2 [file Image_1.jpeg]
